# Pro-inflammatory properties of H-ferritin on human macrophages, ex vivo and in vitro observations

**DOI:** 10.1038/s41598-020-69031-w

**Published:** 2020-07-22

**Authors:** Piero Ruscitti, Paola Di Benedetto, Onorina Berardicurti, Noemi Panzera, Nicolò Grazia, Anna Rita Lizzi, Paola Cipriani, Yehuda Shoenfeld, Roberto Giacomelli

**Affiliations:** 1 Division of Rheumatology, Department of Biotechnological and Applied Clinical Sciences, University of L’Aquila, Delta 6 Building, Via dell’Ospedale, 67100 L’Aquila, Italy; 2 Clinical Pathology Unit, Department of Biotechnological and Applied Clinical Sciences, University of L’Aquila, L’Aquila, Italy; 3 Department of Biotechnological and Applied Clinical Sciences, University of L’Aquila, L’Aquila, Italy; 4 Zabludowicz Center for Autoimmune Diseases, Sheba Medical Center, Tel HaShomer, Israel; 5 Sackler Faculty of Medicine, Tel-Aviv University, Tel Aviv, Israel; 6 Laboratory of the Mosaics of Autoimmunity, Saint Petersburg State University, Saint Petersburg, Russia

**Keywords:** Innate immune cells, Rheumatology

## Abstract

Ferritin is an iron-binding molecule, which comprises 24 subunits, heavy (FeH) and light (FeL) subunits, suggested to have a pathogenic role by the ‘hyperferritinemic syndrome’. In this work, we tested (1) FeH and FeL in bone marrow (BM) and sera in patients with macrophage activation syndrome (MAS); (2) pro-inflammatory effects of ferritin, FeL, and FeH on macrophages; (3) ability of FeH-stimulated macrophages to stimulate the proliferation of peripheral blood mononuclear cells (PBMCs); (4) production of mature IL-1β and IL-12p70 in extracellular compartments of FeH-stimulated macrophages. Immunofluorescence analysis and liquid chromatography mass spectrometry (LC–MS/MS) based proteomics were performed to identify FeL and FeH in BM and sera, respectively, in the same patients. Macrophages were stimulated with ferritin, FeH, and FeL to assess pro-inflammatory effects by RT-PCR and western blot. The proliferation of co-cultured PBMCs with FeH-stimulated macrophages was tested. Immunofluorescence showed an increased FeH expression in BMs, whereas LC–MS/MS identified that FeL was mainly represented in sera. FeH induced a significant increase of gene expressions of IL-1β, IL-6, IL-12, and TNF-α, more marked with FeH, which also stimulated NLRP3. FeH-stimulated macrophages enhanced the proliferation of PBMCs. The ELISA assays showed that mature form of IL-1β and IL-12p70 were increased, in extracellular compartments of FeH-stimulated macrophages. Our results showed FeH in BM biopsies of MAS patients, whereas, LC–MS/MS identified FeL in the sera. FeH showed pro-inflammatory effects on macrophages, stimulated NLRP3, and increased PBMCs proliferation.

## Introduction

Adult-onset Still’s disease (AOSD) is an inflammatory disease characterised by high spiking fevers, arthritis, evanescent skin rash, and a typical increase of serum ferritin levels^1,2^. AOSD is considered a multigenic autoinflammatory disease at the “crossroads” of autoinflammatory and autoimmune diseases, considering its complex pathogenesis, which involves both arms of the immune system^3^. The aberrant activation of the immune system leads to production and release of pro-inflammatory cytokines including interleukin (IL)-1β, IL-6, IL-18, interferon (IFN)-γ and tumor necrosis factor (TNF-α)^1–3^, which represent common therapeutic targets^4,5^. Patients with AOSD may experience several life-threatening complications, mostly macrophage activation syndrome (MAS), a hyperinflammatory syndrome, with high mortality rate^6,7^. Considering the frequent association between these two diseases, it has been suggested that MAS and AOSD may be anchored to the same disease spectrum, representing more severe and milder form, respectively^8^. Continuous high fever, hepatosplenomegaly, severe peripheral blood cytopenia, high serum ferritin levels, and haemophagocytosis by activated macrophages in bone marrow (BM) are typical features of these patients^9^. A multi-layer MAS pathogenic model has been suggested, comprising genetic factors, pro-inflammatory milieu associated with the underlying rheumatic disease, trigger factors and the uncontrolled activation of macrophages and T cells may lead to the development of cytokine storm and MAS^10,11^. The activation of cytotoxic CD8 + T lymphocytes, defects in granulocyte-mediated cytotoxicity, may enhance antigen presentation, and the consequent repeated stimulation of Toll-like receptors would determine the massive production of pro-inflammatory cytokines, with uncontrolled activation and expansion of monocytes and macrophages^12,13^.

Recently, it has been proposed that AOSD, MAS, but also catastrophic anti-phospholipid syndrome (cAPS), and septic shock, which are all characterised by very elevated levels of serum ferritin, would be included under a common umbrella, the so-called ‘hyperferritinaemic syndrome’^14^. Ferritin is an iron storage protein composed of 24 subunits, heavy subunits (FeH) and light (FeL) subunits, which are differently represented in tissues^15,16^. Although the main modulator of this molecules is the iron availability, ferritin synthesis may also be regulated by different inflammatory cytokines^17^, thus suggesting a possible role in inflammation^18,19^. On these bases, in this work, we aimed at assessing the presence of FeH and FeL in bone marrow (BM) biopsies of patients with AOSD and complicated with MAS. In the same patients, we tested what was the prevalent subunit of ferritin, either FeH or FeL, in sera, to analyse possible discrepancy between BM and peripheral blood. Furthermore, we evaluated the effects of ferritin, FeH, and FeL on human macrophages, assessing pro- and anti-inflammatory cytokines, and expression of NLRP3. Finally, we checked the ability of macrophages, which were stimulated with these molecules, to enhance or not the proliferation of peripheral blood mononuclear cells (PBMCs) and the production of mature IL-1β and IL-12p70 in extracellular compartments of FeH-stimulated macrophages.

## Results

### Increased expression of FeH in BM of patients with AOSD and MAS

**Figure 1:**
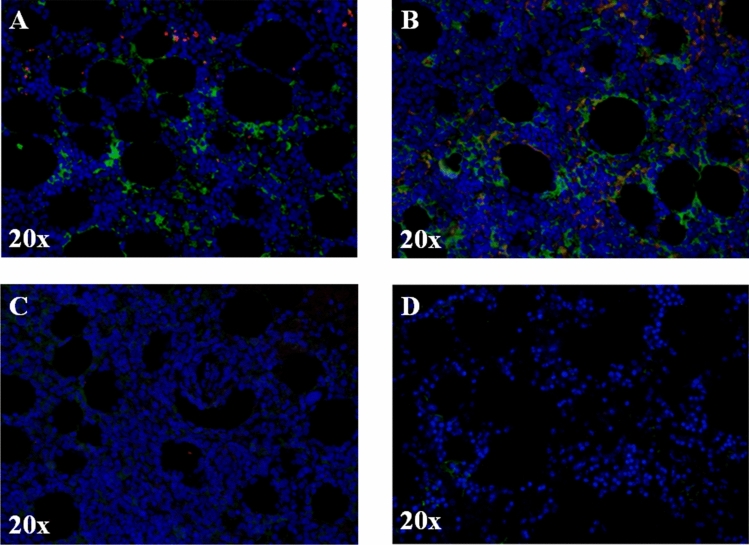
FeH is more represented than FeL in BM biopsies of AOSD complicated with MAS. (**A**, **B**) Immunofluorescence staining of BM biopsies of patients with AOSD complicated with MAS. The representative images of two patients (**A**, **B**) and two HCs (**C**) and (**D**) coloured with FeH (green) and FeL (red) are shown. The intensity of FeH expression is higher, when compared with FeL, since it is more represented. Pictures are representative of all experiments. Original magnification 20×.

As shown in Fig. 1, our results showed that FeH was more represented than FeL in BM biopsies of patients with AOSD and MAS. A granular morphological pattern of both FeH and FeL immunoreactivity was observed, including cytoplasmic and extracellular localisation. Furthermore, in BM samples of patients with AOSD complicated by MAS, both FeH and FeL expression were more represented than those in healthy controls (HCs).

### Liquid chromatography mass spectrometry identification of FeL in sera of patients with AOSD and MAS

In the same patients, who underwent BM biopsies, liquid chromatography mass spectrometry (LC–MS/MS) analysis has successfully identified both FeH and FeL along with many other proteins from the assessed bands. The samples were analysed using All Taxonomy in Uniprot and also an in-house curated database containing the FeH protein sequence only. Following analysis against All Taxonomy at 95% CI probability, a mixed population of proteins was identified from each assessed band, a filtering of the data to highlight the protein of interest was performed. Although the sample was tested for FeH, the best matched search from the database was for the FeL. Following database analysis against All Taxonomy in Uniprot, the sequence coverage of the FeL form was consistent across the assessed samples and ranged from 47 to 58% coverage. The number of total peptides detected in each sample varied from 27 to 36. The number of peptides matching to the sequence of the FeH was very low which was not expected. To see if this had an effect on the dataset, an in-house database was curated containing only the FASTA file of the FeH protein of interest and searched again. This new search increased the number of peptides matched to the FeH form when compared to the Uniprot search but it was still not as convincing as the peptide matching to the FeL. Taking together all these observations, LC–MS/MS has sequenced and identified ferritin in both H- and L-forms from the assessed bands. The analysis of database suggested that the dominant form, after stringent probability matching, was FeL and not FeH, as observed in BM biopsies, in the same patients.

### Macrophages genes expression after stimulation with ferritin, FeH, FeL

**Figure 2:**
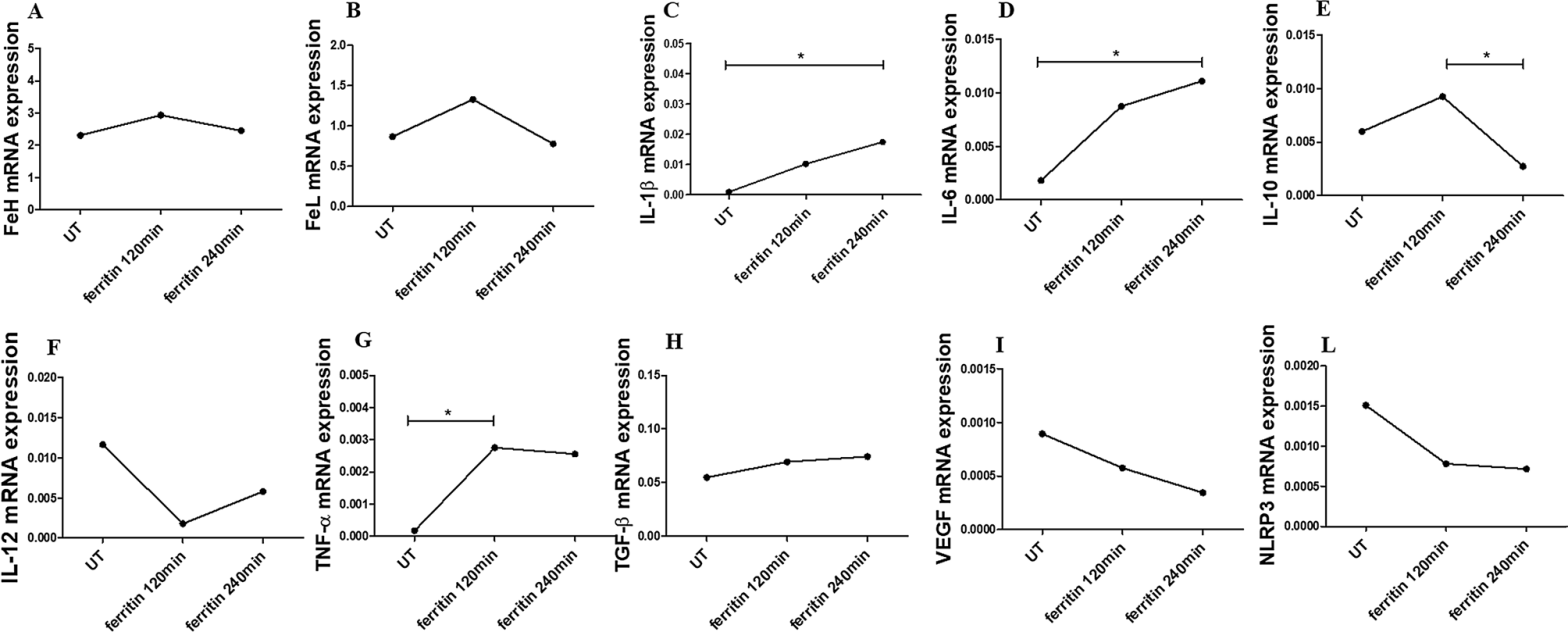
The effects of ferritin on gene expressions of macrophages. (**A**–**L**) qRT-PCR of FeH (**A**), FeL (**B**), IL-1β (**C**), IL-6 (**D**), IL-10 (**E**), IL-12 (**F**), TNF-α (**G**), TGF-β (**H**), VEGF (**I**), NLRP3 (**L**). The ferritin stimulation of macrophages, for both 120 and 240 min, does not change the gene expressions of FeH, FeL, IL-10, IL-12, TGF-β, VEGF and NLPR3. The gene expressions of IL-1β, IL-6 and TNF-α significantly increase following ferritin stimulation. Any single dot, in the figure, represents the median of triplicate experiments (* = p = 0.02).

After stimulation of macrophages with ferritin, for 120 and 240 min, respectively, the mRNA expression of FeH, FeL, IL-10, IL-12, TGF-β, VEGF and NLRP3 remained unchanged, when compared with UT cells. The ferritin significantly increased the mRNA levels of IL-1β, IL-6 and TNF-α genes, when compared with UT cells [IL-1β mRNA levels in ferritin treated cells for 240 min 0.017 (0.0011–0.94) vs IL-1β mRNA levels in UT treated cells 0.00092 (8.547e − 5 to 0.0025), p = 0.02; IL-6 mRNA levels in ferritin treated cells for 240 min 0.011 (0.0026–0.022) vs IL-6 mRNA levels in UT treated cells 0.0018 (0.00021–0.0054), p = 0.02; TNF-α mRNA levels in ferritin treated cells for 120 min 0.0027 (1.07e − 5 to 0.0084) vs TNF-α mRNA levels in UT treated cells 0.00017 (1.470e − 6 to 0.00040, p = 0.02] (Fig. 2).

**Figure 3:**
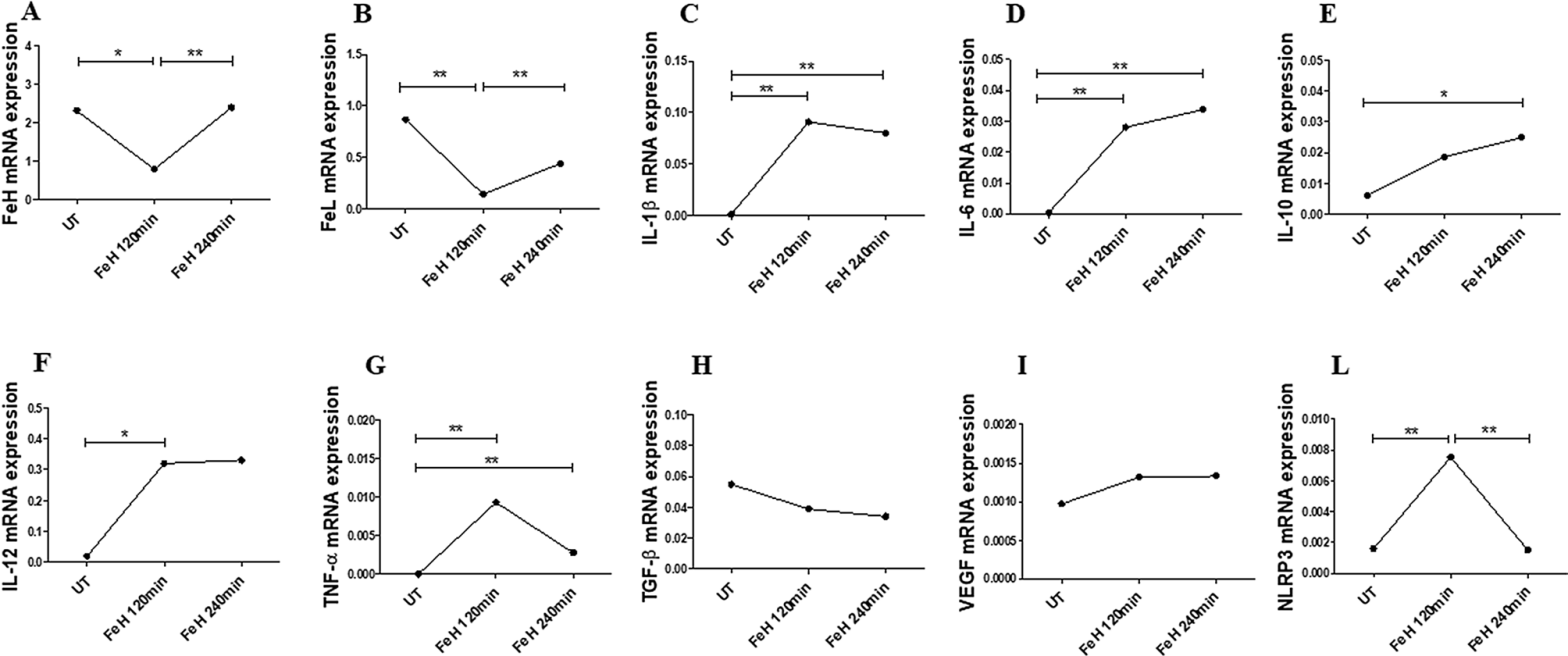
The effects of FeH on gene expressions of macrophages. (**A**–**L**) qRT-PCR of FeH (**A**), FeL (**B**), IL-1β (**C**), IL-6 (**D**), IL-10 (**E**), IL-12 (**F**), TNF-α (**G**), TGF-β (**H**), VEGF (**I**), NLRP3 (**L**). The FeH stimulation of macrophages for both 120 and 240 min, does not change the gene expression of TGF-β and VEGF. The gene expressions of FeH and FeL significantly decrease after FeH stimulation, for 120 min. The gene expressions of IL-1β, IL-6, IL-10, TNF-α, IL-12 and NLRP3 significantly increase after FeH stimulation. Any single dot, in the figure, represents the median of triplicate experiments (* = p = 0.02; ** = p ≤ 0.008).

Furthermore, as reported in Fig. 3, after stimulation with FeH, for 120 and 240 min, respectively, the mRNA expression of TGF-β and VEGF remained unchanged, when compared to UT cells. Concerning FeH and FeL genes, the levels of mRNA expression significantly decreased after 120 min of FeH stimulation, when compared with UT cells, but after 240 min the levels of mRNA expressions were returned comparable to those of UT cells [FeH mRNA levels, in FeH treated cells for 120 min 0.79 (0.60–1.51) vs FeH mRNA levels, in UT treated cells 2.31 (0.87–3.73), p = 0.02; FeL mRNA levels, in FeH treated cells for 120 min 0.14 (0.069–0.31) vs FeL mRNA levels, in UT treated cells 0.87 (0.30–1.53), p = 0.008]. The FeH stimulation, for both 120 and 240 min, induced a significant increase of mRNA levels of expressions of IL-1β, IL-6, IL-10, IL-12 and TNF-α, when compared with UT cells [IL-1β mRNA levels in FeH treated cells for 120 min 0.09 (0.049–0.10); IL-1β mRNA levels in FeH treated cells for 240 min 0.08 (0.03–0.18) vs IL-1β mRNA levels in UT treated cells 0.0009 (0.0006–0.005), p = 0.002; IL-6 mRNA levels in FeH treated cells for 120 min 0.028 (0.016–0.03); IL-6 mRNA levels in FeH treated cells for 240 min 0.03 (0.01–0.08) vs IL-6 mRNA levels in UT treated cells 0.0002 (3.99e − 5 to 0.005), p = 0.002; IL-10 mRNA levels in FeH treated cells for 240 min 0.024 (0.019–0.044) vs IL-6 mRNA levels in UT treated cells 0.005996 (0.0032–0.0093), p = 0.002; TNF-α mRNA levels in FeH treated cells for 120 min 0.009300 (0.0002–0.02); TNF-α mRNA levels in FeH treated cells for 240 min 0.002 (0.002–0.01) vs TNF-α mRNA levels in UT treated cells 8.54e-5 (7.0e − 6 to 0.0004), p = 0.008; IL-12 mRNA levels in FeH treated cells for 120 min 0.32 (0.09–1.4) vs IL-12 mRNA levels in UT treated cells 0.01 (0.006–0.5), p = 0.02]. FeH stimulation significantly increased mRNA levels of expression of NLRP3 gene after 120 min, when compared with UT cells, although after 240 min these levels were comparable to UT cells [NLRP3 mRNA levels, in FeH treated cells for 120 min 0.007 (0.007–0.03) vs NLRP3 mRNA levels, in UT treated cells 0.0015 (0.001–0.003), p = 0.002].

Additionally, we analysed the effects of FeL stimulation of human macrophages, for both 120 and 240 min. The results showed that the mRNA expression of all analysed genes remained unchanged when compared with UT cells (Additional Material 1).

### Macrophages intra-cellular proteins expression after stimulation with ferritin and FeH

**Figure 4:**
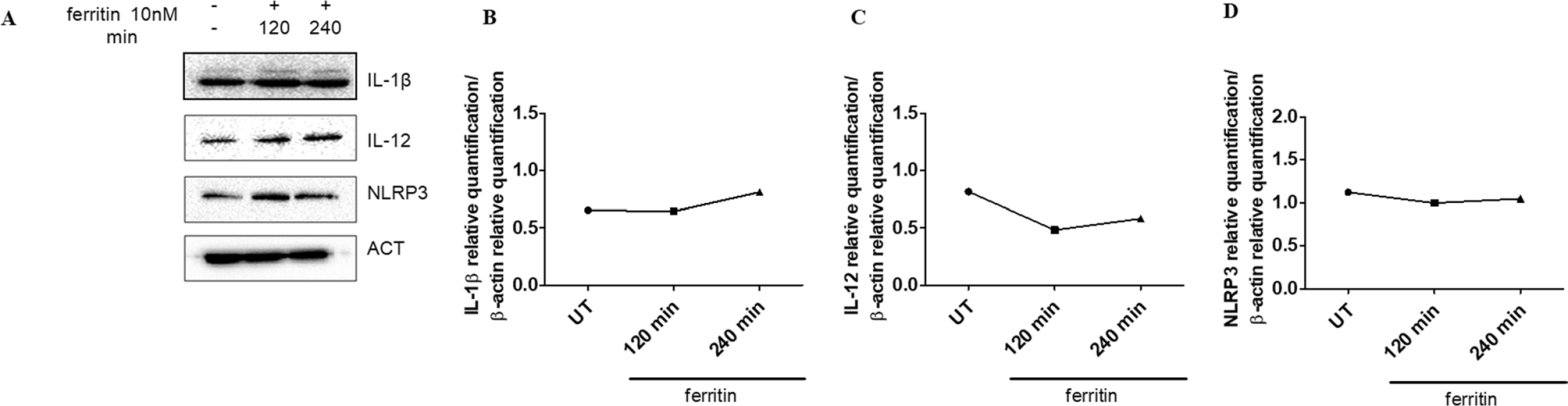
The effects of ferritin on intracellular protein expressions of macrophages. (**A**) Western blot analyses of IL-1β, IL-12 and NLPR3, pictures are representative of all the experiments. The grouping of gels/blots cropped from different parts of the same gel. No high-contrast (overexposure) of blots was performed. (**B**–**D**) The densitometry analysis of western blot shows that the ferritin stimulation of macrophages, for both 120 and 240 min, does not change the protein expressions of IL-1β (**B**), IL-12 (**C**) and NLPR3 (**D**). Any single dot, in the figure, represents the median of triplicate experiments.

**Figure 5:**
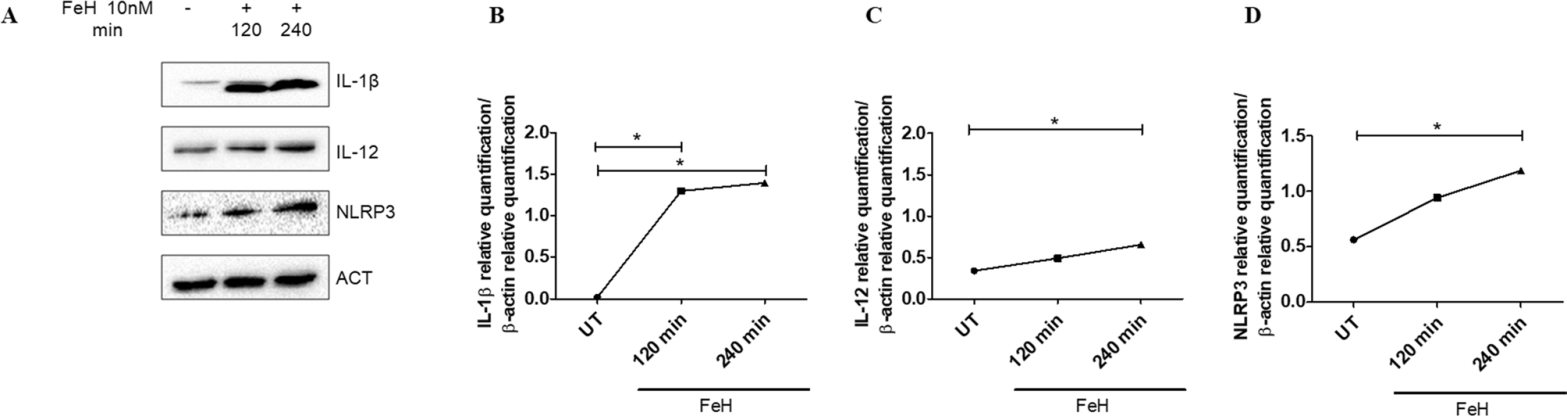
The effects of FeH on intracellular protein expressions of macrophages. (**A**) Western blot analyses of IL-1β, NLPR3 and IL-12, pictures are representative of all the experiments. The grouping of gels/blots cropped from different parts of the same gel. No high-contrast (overexposure) of blots was performed. (**B**–**D**) The densitometry analysis of western blot shows that the FeH stimulation of macrophages for 120 and 240 min, significant increases the protein expression of IL-1β (**B**), IL-12 (**C**) and NLPR3 (**D**). Any single dot, in the figure, represents the median of triplicate experiments (* = p = 0.049).

After stimulation of macrophages with ferritin, for 120 and 240 min, the proteins expression of IL-1β, NLRP3 and IL-12 remained unchanged when compared to UT cells (Fig. 4). Interestingly, when macrophages were stimulated with FeH for 240 min, the levels of proteins expression of IL-1β, NLRP3 and IL-12 were significantly increased when compared with UT cells (p = 0.049) (Fig. 5). Finally, we tested macrophages proteins expression of IL-6 and TNF-α, after stimulation with ferritin and FeH, for 120 and 240 min, but non-significant results were obtained (data not shown).

### Macrophages extracellular protein expression after stimulation with FeH

**Figure 6:**
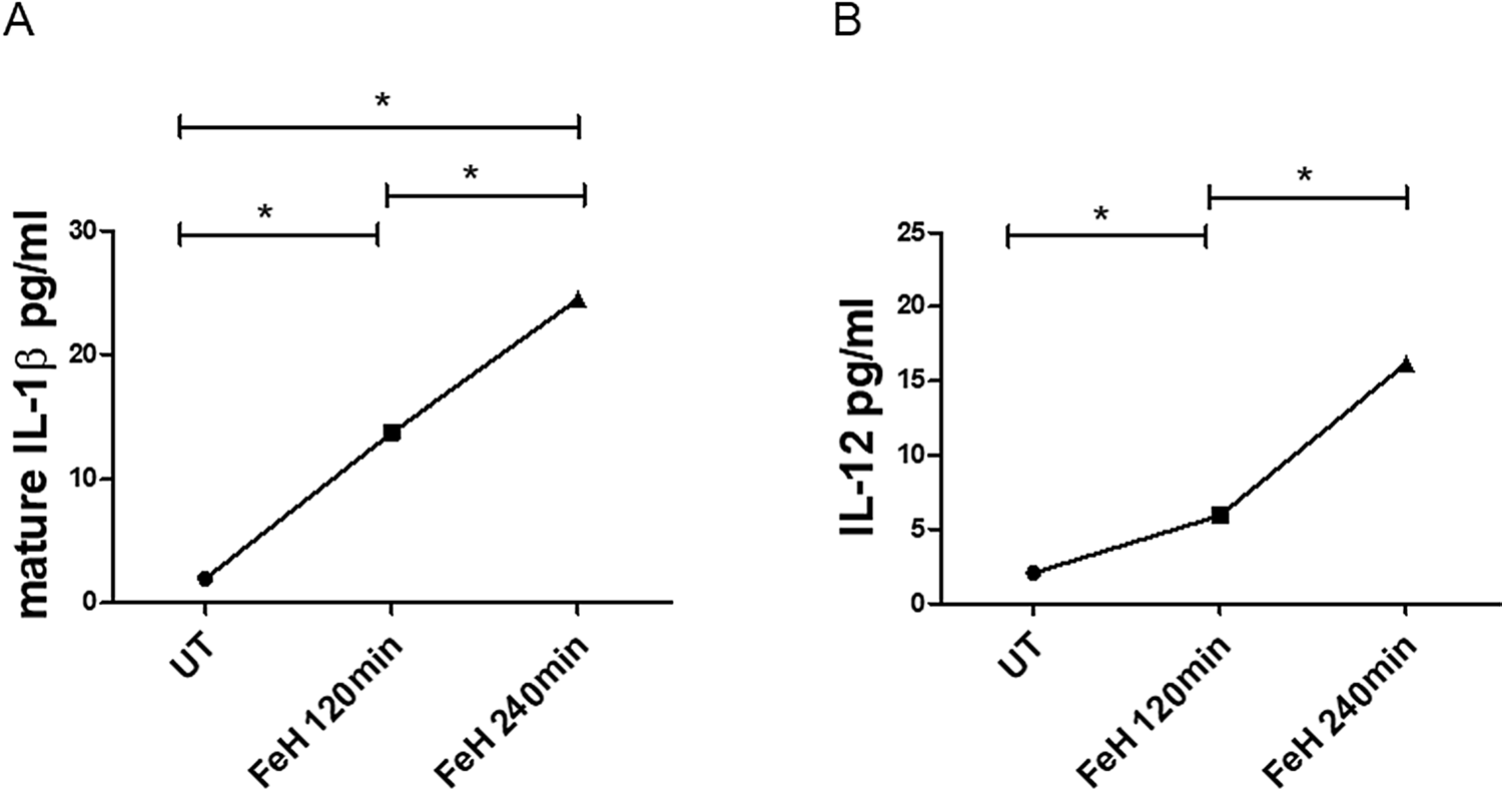
Effects of FeH on extracellular protein expression of macrophage. (**A**, **B**) ELISA assays. Mature IL-1β (**A**) and IL-12p70 (**B**) levels on supernatants from macrophages untreated (UT) or stimulated with FeH for 120 min and 240 min. The levels of mature form of IL-1β and IL-12p70, after stimulation with FeH for both 120 min and 240 min, were significantly increased when compared to UT cells. Any single dot, in the figure, represents the median of triplicate of the same experiment experiments (* = p < 0.05).

The ELISA assay showed that the levels of mature form of IL-1β, following stimulation with FeH for both 120 min and 240 min, were significantly increased when compared with UT cells [mature IL-1β levels in FeH treated cells for 120 min 13.73 pg/ml (13.49–13.96) vs mature IL-1β levels in UT cells 1.96 pg/ml (1.68–2.14); p = 0.048. Mature IL-1β levels in FeH treated cells for 240 min 24.49 pg/ml (22.83–26.00) vs mature IL-1β levels in UT cells 1.96 pg/ml (1.68–2.14); p = 0.047] (Fig. 6A). Similarly, IL-12p70 levels, following stimulation with FeH for both 120 min and 240 min, were significantly increased when compared to UT cells [IL-12p70 levels in FeH treated cells for 120 min 5.93 pg/ml (4.87–10.04) vs IL-12p70 levels in UT cells 2.06 pg/ml (2.06–3.85); p = 0.03; IL-12p70 levels in FeH treated cells for 240 min 16.15 pg/ml (7.85–20.2) vs IL-12p70 levels in UT cells 2.06 pg/ml (2.06–3.85) p = 0.03] (Fig. 6B).

### Stimulation of macrophages with FeH increased PBMCs proliferation

**Figure 7:**
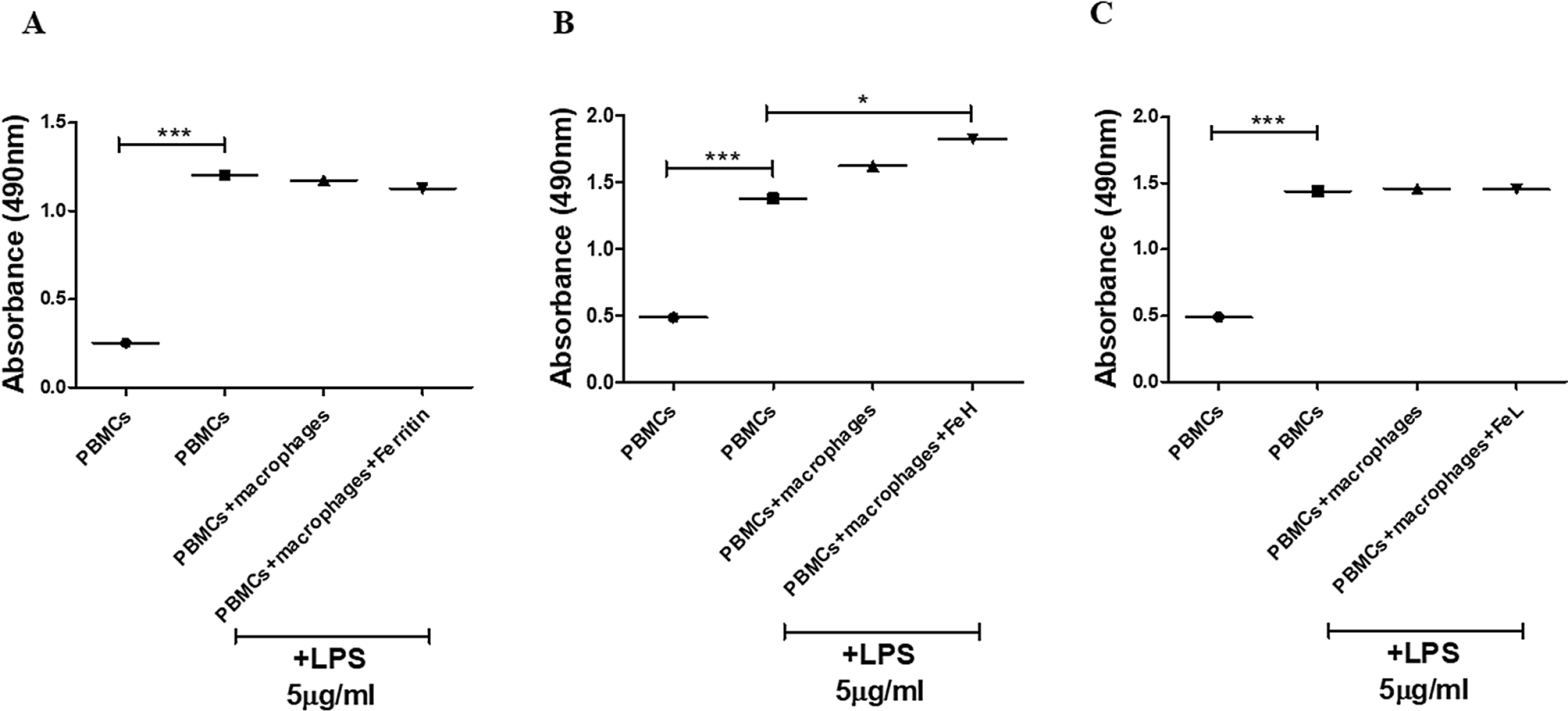
Effects of ferritin, FeH and FeL on the proliferation of PBMCs co-cultured with macrophages. (**A**–**C**) PBMCs proliferation using MTS assay. (**A**) The picture shows the absorbance of untreated PBMCs (circle), PBMCs treated with LPS (square), PBMCs treated with LPS and co-cultured with untreated macrophages (triangle up), PBMCs stimulated with LPS and co-cultured with macrophages stimulated with ferritin (triangle down). Any single dot, in the figure, represents the median of triplicate experiments (* = p = 0.02; *** = p < 0.0001). (**B**) The picture shows the absorbance of untreated PBMCs (circle), PBMCs treated with LPS (square), PBMCs treated with LPS and co-cultured with untreated macrophages (triangle up), PBMCs stimulated with LPS and co-cultured with macrophages stimulated with FeH (triangle down). Any single dot, in the figure, represents the median of triplicate experiments (* = p = 0.02; *** = p < 0.0001). (**C**) The picture shows the absorbance of untreated PBMCs (circle), PBMCs-treated with LPS (square), PBMCs treated with LPS and co-cultured with untreated macrophages (triangle up), PBMCs stimulated with LPS and co-cultured with macrophages stimulated with FeL (triangle down). Any single dot, in the figure, represents the median of triplicate experiments (*** = p = 0.0002).

Healthy human PBMCs proliferation was assessed by co-culturing LPS-activated PBMCs with macrophages with and without stimulation with FeH. LPS induced a significant increase of PBMCs proliferation after 7 days of stimulation, when compared with UT-PBMCs [absorbance 0.47 (0.20–0.53) in UT-PBMCs vs 1.4 (0.68–1.71) in PBMCs + LPS, p < 0.0001]. The co-culture of LPS-treated-PBMCs with UT-macrophages did not promote the proliferation of PBMCs, whereas the stimulation of macrophage with FeH, changed the macrophages behaviour and stimulated a significant increase of PBMCs proliferation [absorbance 1.8 (0.97–2.0) in PBMCs + LPS + macrophage + FeH vs 1.4 (0.98–1.71) in PBMCs + LPS, p = 0.02] (Fig. 7B). On the contrary, the stimulation of macrophages with ferritin or FeL did not induce an increase of PBMCs proliferation, as shown in Fig. 7A,C.

## Discussion

In this work, the results showed the presence of FeH in BM biopsies of AOSD patients complicated with MAS patients, whereas FeL was the predominant form in the sera of those. Furthermore, pro-inflammatory effects of FeH on human macrophages were observed in vitro, increasing pro-inflammatory cytokines and NLRP3. Finally, FeH-treated macrophages enhanced the proliferation of co-cultured PBMCs. Taking together all these results and considering that AOSD and MAS could be included in the so-called “hyperferritinaemic syndrome”^14^, our data could reinforce the hypothesis that higher levels of ferritin may not only be considered a consequence or an epiphenomenon of the inflammation, but it may actively play a role in pathogenic mechanisms of those diseases, thus enhancing the inflammatory burden.

Our results showed that FeH was more represented than FeL in BM biopsies of patients with AOSD and MAS, as previously reported in affected tissues^18,19,23,24^. Conversely, in sera of these patients, FeL was the predominant form, as reported by LC–MS/MS*.* Based on that discrepancy, we tested the inflammatory properties of ferritin, FeH, and FeL on human macrophages, and we observed that ferritin and, as particularly, FeH induced the expression of pro-inflammatory cytokines. Specifically, an increased gene expression of IL-1β, IL-6, IL-12, and TNF-α was observed. In this context, pro-inflammatory cytokines are largely overexpressed in patients with AOSD complicated with MAS^25–27^ and may induce preferentially the expression of FeH, via FER2. The latter, after activation, stimulates the synthesis of FeH and the production of many pro-inflammatory cytokines, perpetuating a vicious pathogenic inflammatory circle^28–32^. In addition, the assessment of protein expressions showed the stimulation of macrophages with FeH induced a significant increase of IL-1β, and IL-12, when compared to UT cells, whereas ferritin and FeH did not. These discrepancies among ferritin, FeH, and FeL could be related to a different effect of these molecules on macrophages, as observed for gene expression. Furthermore, the production of mature form of IL-1β, in the extracellular compartment, was induced by FeH stimulation on macrophages. This finding could suggest a specific pathogenic link between FeH and IL-1β, which is a crucial mediator in AOSD and MAS, also because of clinical usefulness of IL-1 inhibition in those patients^33,34^. In fact, multiple lines of evidence suggested the efficacy of IL-1 inhibition in the context of the hyperferritinaemic syndrome^35–37^. Furthermore, the lack of confirmation of protein expression of both IL-6 and TNF-α could reinforce this hypothesis. Moreover, the efficacy of IL-6 and TNF-α inhibition reported conflicting results^5,38^. Additionally, paralleling with IL-1β, FeH induced a significant expression of NLRP3, a cytosolic innate immune signalling receptor, which is the main factor associated with the maturation and production of this cytokine^39^. Interestingly, our data could suggest a vicious cycle by FeH, as a further stimulator of NLRP3, since it could be an additional danger signal in triggering this factor. The activation of NLRP3 begins with the recognition of the danger or stressor, pathogen/damage-associated molecular patterns (PAMPs/DAMPs), by the sensor pattern recognition receptors (PRRs)^40^. Once activated, NLRP3 nucleates the assembly of an inflammasome, by interacting with an adaptor apoptosis speck-like protein (ASC), recruits and activates procaspase-1 to generate active caspase-1 and then converts the cytokine precursors pro-IL-1β into mature and biologically active IL-1β^41,42^. After that, a series of inflammatory mechanisms and pyroptotic cell death are triggered^43^. Taking together all these observations and considering its involvement in AOSD^44,45^, the direct stimulation of NLRP3 by FeH could provide further insights to the pathogenesis of these diseases, linking the typical hyperferritinemia with the production of a crucial pathogenic mediator. Additionally, FeH induced a significant expression of intracellular IL-12 as well as promoted its release in the extracellular compartment. It has been reported that IL-12 is a pro-inflammatory cytokine produced by dendritic cells, macrophages and B cells in response to microbial pathogens^46^. On this basis, we could speculate that IL-12, increased in our experimental conditions, could play a pro-inflammatory role. Interestingly, it has been shown that after over-expression of IL-12, the phenotype of M2 macrophages could be re-directed to that of M1-like macrophages^47^. Although it is presently known that functional polarization of macrophages is an over-simplified description of macrophage heterogeneity and plasticity, two classical different phenotypes of macrophages were described, considered the end-stage phenotypes of a continuum of functional states, classically activated (or inflammatory) macrophages (M1) and the other alternatively activated (or wound-healing) macrophages (M2)^48,49^. In this context, a differential cytokine production is a key feature of polarized macrophages^49^. The M1 phenotype is typically IL-12^high^ and IL-10^low^, whereas M2 macrophages are typically IL-10^high^ and IL-12^low^^50^. Furthermore, the stimulation of macrophages with FeH, enhanced the proliferation of co-cultured PBMCs. Taking together these results and previous observations^51–54^, it could be possible to hypothesize that the stimulation with FeH could orientate the macrophages toward an M1 phenotype, suggesting the need of further studies to entirely clarify these issues. In addition, in inflammatory infiltrate of AOSD and MAS, a specific subset of macrophages was reported, displaying a specific CD68/H-ferritin phenotype expressing IL-12, which cannot be observed in normal tissues^19,23^. Finally, considering that the protein expressions did not confirmed the gene expressions observed following stimulation with ferritin on pro-inflammatory mediators, it could be possible to attribute the pro-inflammatory effects to FeH subunits of the ferritin.

In this study, the effects FeL on pro-inflammatory cytokines were also tested, but non-significant results were obtained. In fact, it has been recently reported that a compensatory increase of FeL, after deletion of FeH, could reduce the cytokine levels, the multi-organ dysfunction and the mortality in a murine model of sepsis^55^. In fact, an inhibitory action of FeL was shown on NF-kB activation, a key signalling pathway which is implicated in the pathogenesis of sepsis but also of other inflammatory diseases^55^. On the contrary, a stimulatory effect of FeH on NF-kB was described acting as a pro-inflammatory cytokine on hepatic stellate cells^56^. Additionally, it has been reported that FeH could modulate macrophage response to immune stimuli^57^. Taking together all these findings, it is possible to suggest that the pro-inflammatory effects of ferritin could be mainly attributed to FeH than FeL, suggesting also a possible therapeutic target to be investigated in future specific designed studies.

Taking together all these data, a contributory role of ferritin as a pathogenic mediator rather than being a product of inflammation could be suggested, but, additionally, ferritin is proposed to be a biomarker for the disease in early diagnosing and in monitoring the clinical response to therapies^58^. In fact, hyperferritinemia, may identify a more aggressive subset of diseases, and, as observed in cAPS and MAS, its reduction, after treatment, is associated with a lower mortality^59–61^. In addition, recent evidence from coronavirus disease 2019 (COVID-19), identified hyperferritinemia and IL-6 as predictors of poor prognosis, suggesting that the mortality of these patients is related to a hyper-inflammatory process^62–64^. This finding could thus hypothesize the inclusion of COVID-19, at least for a more severe subset of patients, in the “hyperferritinaemic syndrome”, since sharing pathogenic mechanisms, clinical features, and possibly therapeutic targets.

In spite of suggesting possible pro-inflammatory properties of ferritin and particularly of FeH, our work is affected by some limitations, such as the relative low number of assessed patients, which could limit the external validity. However, it must be pointed out that AOSD and MAS are very rare diseases and it is very challenging to get matched BM biopsies and peripheral blood samples. An additional challenge is the severity of the diseases rapidly evolving into a life-threatening clinical picture, complicating even more the collection of biologic samples from affected patients. Taking together these observations, further confirmatory and mechanicistic studies are needed to fully elucidate these issues.

In conclusion, pro-inflammatory effects of FeH on human macrophages could be suggested, since it increased the expression of the pro-inflammatory cytokines and NLRP3, and enhanced the proliferation of co-cultured PBMCs. Considering these results and previous observations^51–53^, it could be possible to hypothesize that the stimulation with FeH could orientate the macrophages toward an M1 phenotype, suggesting the need of further studies to entirely clarify this issue. Taking together all these results and considering that AOSD and MAS could be included in the so-called “hyperferritinaemic syndrome”, our data could reinforce the hypothesis that higher levels of ferritin may not only be considered a consequence of the inflammation, but it may actively play a role in the pathogenic mechanisms of those diseases enhancing the inflammatory burden. In addition, given that these pro-inflammatory effects could be mainly attributed to FeH, it could be also possible to speculate a possible new therapeutic target to be tested to improve the management of these patients.

## Patients and methods

### Patients

Four patients with AOSD complicated with MAS were assessed at the time of diagnosis, collecting BM biopsies and sera, which were analysed at the same time. All these patients were admitted to the Rheumatology Clinic of L’Aquila University, Italy, and fulfilled the diagnostic criteria proposed by for AOSD and MAS in rheumatic diseases^20–22^. In this study, we also evaluated 2 BM biopsies derived from BM-donors, used as HCs. The local Ethics Committee approved the study (*ASL1 Avezzano-Sulmona-L’Aquila*, L’Aquila, Italy, protocol number 0122353/17) that was performed according to Good Clinical Practice guidelines and Declaration of Helsinki. Each patient provided informed consent for purposes of the study.

### Histological analysis of biopsies

Four BM biopsies were evaluated, which derived from patients with AOSD complicated by MAS. The immunofluorescence analysis was performed on paraffin sections (thickness 3 µm) and antigen retrieval was carried out using target retrieval solution (DAKO, USA). Samples were stained with anti-FeH, anti-FeL antibodies (Santa Cruz Biotechnology, USA), as reported previously^18,19^. The immunoreaction was revealed by using a secondary antibody (Alexa fluor, Sigma-Aldrich, USA). Cell nuclei were visualized using 4′,6-diamidino-2-phenylindole. The fluorescence was assessed by using an Olympus BX53 fluorescence microscope.

### Enzymatic digestion

Four sera of patients with AOSD were mixed with 50 mM sodium acetate (Sigma Aldrich, Germany), Ph4.8, heated at 70 °C for 10 min and centrifugated at 15000 g for 30 min at 4 °C. Maintaining the solution at PH 5.2 (4 °C), ammonium sulfate (Sigma Aldrich, Germany) (50% of saturation) was added and the final solution was centrifuged at 15000g for 30 min at 4 °C. The final pellet was re-suspended in PBS (PH 7.0). Proteins contained in the final solution and recombinant FeH (Abcam, UK) were separated by 15% SDS-PAGE gel. The band with a molecular weight 18–25 kDa, with the same weight of recombinant FeH were collected. In-gel reduction, alkylation and digestion with trypsin were performed on the four gel bands before to a subsequent analysis by a mass spectrometry, as previously reported^65^.

### LC–MS/MS

Peptides were extracted from the gel pieces, the peptides were resolved by reversed phase chromatography and the evaluate was ionised by electrospray ionisation using an Orbitrap Velos Pro (Thermo Fisher Scientific, UK) operating under Xcalibur v2.2, as previously reported^65^. The report of LC–MS/MS analysis is reported in Additional material 2.

### Database searching

Raw mass spectrometry data were processed into peak list files using Proteome Discoverer (Thermo Fisher Scientific; v2.1). Processed raw data were searched for using the Mascot search algorithm (v2.6; https://www.matrixscience.com) against the Uniprot database with All Taxonomy. The data were also searched against an in-house curated database containing only the FeH protein of interest. The methods detailed in Sects. 2.3, 2.4 and 2.5 were performed by Centre of Excellence for Mass Spectrometry, King’s College London, London, UK. The report of LC–MS/MS analysis is reported in Additional material 2.

### Monocytes isolation and differentiation

Peripheral blood monocytes were obtained from healthy donors by direct isolation using whole blood collected in 1 mM ethylenediaminetetracetic acid (EDTA) (Sigma-Aldrich, USA) and mixed with 50 µl/ml RosetteSep human monocytes enrichment cocktail (Stemcell, USA), according to the manufacturer’s protocol. The derived enriched human monocytes were plated 2 × 10^5^/cm^2^ in Roswell Park Memorial Institute (RPMI) 1,640 Medium (EuroClone, Europe), supplemented with 10% foetal bovine serum (FBS; Gibco, USA), 2 mmol/l l-glutamine (EuroClone, Europe) and 100 U penicillin, 1,000 U streptomycin (Biochrom AG, Germany). Additionally, these cells were cultured for 7 days with 50 ng/ml macrophage colony-stimulating factor (M-CSF) (PromoKine, Germany), changing the medium every 2 days. Cells were incubated at 37 °C in a humidified atmosphere consisting of 5% CO_2_. Purity of cells was assessed by immunofluorescence staining. Briefly, the cells were fixed with 4% paraformaldehyde (EMS, PA), incubated 20 min with protein block (DAKO, USA) and successively with anti-CD14-antibody (Invitrogen, USA) and anti-CD68 antibody (Santa Cruz Biotechnology, USA). The visualization of the anti-CD14-antibody was performed using an Alexa Fluor 488-conjugated (Invitrogen, USA) and the visualization of the anti-CD68-antibody was performed using an Alexa Fluor 555-conjugated (Invitrogen, USA). After counterstained with 4′,6-diamidino-2-phenylindole (DAPI), images were obtained using an Olympus BX53 fluorescence microscope. The number of CD14+ and CD68+ cells was counted to assess their purity. At day 0, before M-CSF stimulation CD14+ cells were 77.2% and CD68+ cells 22.8%, out of the total percentage of cells, respectively. After 7 days of stimulation with M-CSF, CD14+ cells were 15.4% and CD68+ cells 84.6%, out of the total percentage of cells, respectively (Additional Material 3).

### Stimulation of monocytes with ferritin, FeH, and FeL

To establish the optimal concentration of ferritin, FeH and FeL, in our system, a dose/response curve was performed, evaluating the IL-1β mRNA expression. Each experiment was performed in triplicate (data not show). The derived macrophages were cultured in 10% FBS medium, considering the untreated (UT) condition, and supplemented with selected dose of 10 nM ferritin (Mybiosource, USA), 10 nM FeH (Abcam, UK) and 10 nM FeL (Abcam, UK), for 120 and 240 min.

### qRT-PCR analysis

Total RNA was extracted from macrophages, stimulated with ferritin, FeH and FeL employing Allprep DNA/RNA/miRNA universal kit (Qiagen, Germany) and reverse transcribed into complementary DNA (cDNA) with the High Capacity cDNA Reverse transcription kit (Applied Biosystems, USA). The qRT-PCR was performed by using SYBR green kits and Taqman gene expression assay (Applied Biosystems, Netherlands). The qRT-PCR was run in triplicate. Primers were designed based on the reported sequences [Primer bank NCBI; FeH: 5′-TCCTACGTTTACCTGTCCATGT-3′ (forward) and 5′-GTTTGTGCAGTTCCAGTAGTGA-3′ (reverse); FeL: 5′-CAGCCTGGTCAATTTCTACCT-3′ (forward) and 5′-GCCAATTGCCGGAAGAAGTG-3′ (reverse); IL-1β: 5′-AGCTACGAATCTCCGACCAC-3′ (forward) and 5′- CGTTATCCCATGTGTCGAAGAA-3′ (reverse); IL-6: 5′-AATTCGGTACATCCTCGACGG-3′ (forward) and 5′-TTGGAAGGTTCAGGTTGTTTTCT-3′ (reverse); TNF-α: 5′-CCTCTCTCTAATCAGCCCTCTG-3′ (forward) and 5′-GAGGACCTGGGAGTAGATGAG-3′ (reverse); NLRP3: 5′-TGCGATCAACAGGAGAGACC-3′ (forward) and 5′-CGTGCATTATCTGAACCCCAC-3′ (reverse); IL-10: 5′-TCAAGGCGCATGTGAACTCC-3′ (forward) and 5′-GATGTCAAACTCACTCATGGCT-3′ (reverse); TGF-β: 5′-CTAATGGTGGAAACCCACAACG-3′ (forward) and 5′-TATCGCCAGGAATTGTTGCTG-3′ (reverse); VEGF: 5′-AGGGCAGAATCATCACGAAGT-3′(forward) and 5′-GCTGCGCTGATAGACATCCA-3′ (reverse); β-Actin: 5′-CCTGGCACCCAGCACAAT-3′ (forward) and 5′-AGTACTCCGTGTGGATCGGC-3′ (reverse)]. IL-12 and GAPDH gene expression were assessed by commercial Taqman gene expression assay (Hs01073447_m1; Hs02758991_g1, respectively). Results were analysed after 45 cycles of amplification using the ABI 7500 Fast Real Time PCR System.

### Western blot

Macrophages subjected with ferritin and FeH were lysed in lysis buffer (RIPA buffer, Cell Signaling USA) for 10 min and cleared by centrifugation. The protein concentration was calculated by Bicinchoninic Protein Assay kit (EuroClone, Italy). Forty µg of proteins were separated by SDS-PAGE and transferred to nitrocellulose membranes. After blocking in 10% not fat milk in Tris-buffered saline/1% tween 20 (TBS/T), as previously reported^65^, and incubated with the primary antibodies: IL-12 (sc-7925, Santa Cruz Biothecnology, USA), IL-1β (sc-1250, Santa Cruz Biothecnology, USA) and NLRP3 (AMAb90569, Atlas Antibodies, UK). Following, horseradish peroxidase-conjugated secondary antibodies (Cell Signaling, USA) were appropriately used. The detection was performed by Long Lasting Chemiluminescent Substrate (EuroClone, Italy). All the signals were quantified by normalising against a β-actin signal (sc-130656, Santa Cruz Biothecnology, USA). All the antibodies were already used in previous works for western blot analysis^62–66^. Immunoreactive bands were acquired by chemidoc (ImageLab), and quantified by densitometry using NIHimageJ version 1.5 freeware. Both experiments with ferritin and FeH stimulations were performed in triplicate and the blot of each experiment was reported in Additional Materials 4 and 5.

### Cytokine measurement by ELISA

The human macrophages were also treated with FeH for 120 min and 240 min and the supernatant collected. A solid phase sandwich ELISA assay was used to determine the amount of stimulation induced mature-IL-1β (DY201) and IL-12p70 (D1200) production in macrophages according to the manufacturer’s instructions (R&D Systems, USA). The results were expressed as median of the triplicate of the same experiment.

### Proliferation assay of PBMCs

Human PBMCs preparations were obtained, using heparinised blood carefully layered onto Ficoll (density 1.077 g/ml; Fresenius Kabi Norge AS for Axis-Shield PoC AS, Norway) and centrifuged at 800g for 30 min without brake to obtain a density gradient separation. After centrifugation, the mononuclear cells layer was recovered and washed twice with PBS (Corning, USA)^67^. After FeH stimulation for 120 min, macrophages were co-cultured with PBMCs, 2 × 10^6^/ml, in RPMI-1640 (EuroClone, Europe), supplemented with 10% FBS (FBS; Gibco, USA), in the presence of 5 µg/ml lipopolysaccharides (LPS, Sigma-Aldrich, USA) from Escherichia coli for 7 days. PBMCs alone, without LPS stimulation were used as a control. After LPS stimulation, equal volume of PBMCs were harvested and proliferation were evaluated by the MTS colorimetric technique, using CellTiter 96 Aqueous cell proliferation assay (Promega, USA), according to the manufacturer’s protocol. The MTS tetrazolium compound was bio-reduced by metabolically active cells to a coloured formazan product that was soluble in the tissue culture medium. In this assay, the quantity of formazan product formed is directly proportional to the number of proliferating cells in the cultures. Absorbance was recorded at 490 nm by using a microplate reader (Labda 25 UV/VIS spectrometer, PerkinElmer)^68^.

### Statistical analysis

GraphPad Prism 5.0 software was used for statistical analyses. Results were expressed as median (range), due to the non-parametric distribution of our data. The Mann–Whitney U test was used as appropriate for analyses, as previously performed^66^. Statistical significance was expressed by a p value < 0.05.

## Supplementary information

Supplementary Information

## Data Availability

All data relevant to the study are included in the article or uploaded as Supplementary Information.
