# Supplementary material for: Pro-inflammatory properties of H-ferritin on human macrophages, ex vivo and in vitro observations
Source: Sci Rep. 2020 Jul 22;10:12232. doi: 10.1038/s41598-020-69031-w (PMC7376151; doi:10.1038/s41598-020-69031-w)

Title page

-Title

Pro-inflammatory properties of H-ferritin on human macrophages, *ex vivo* and *in vitro* observations

-Running Title

Pro-inflammatory properties of H-ferritin

-Authors

Piero Ruscitti[§1], Paola Di Benedetto[§2], Onorina Berardicurti[1], Noemi Panzera[2], Nicolò Grazia[2], Anna Rita Lizzi[3], Paola Cipriani[1], Yehuda Shoenfeld[4,5,6], Roberto Giacomelli[1]

[§]: contributed equally to this work

-Affiliations

[1]: Division of Rheumatology, Department of Biotechnological and Applied Clinical Sciences, University of L'Aquila, L'Aquila, Italy.

[2]: Clinical Pathology Unit, Department of Biotechnological and Applied Clinical Sciences, University of L'Aquila, L'Aquila, Italy.

[3]: Department of Biotechnological and Applied Clinical Sciences, University of L'Aquila, L'Aquila, Italy.

[4]: Zabludowicz Center for Autoimmune Diseases, Sheba Medical Center, Tel HaShomer, Israel.

[5]: Sackler Faculty of Medicine, Tel-Aviv University, Tel Aviv, Israel.

[6]: Laboratory of the Mosaics of Autoimmunity, Saint Petersburg State University, Russia.

-Corresponding author

Piero Ruscitti, MD, PhD, Division of Rheumatology, Department of Biotechnological and Applied Clinical Sciences, University of L'Aquila, Delta 6 Building, Via dell'Ospedale, 67100, L'Aquila, Italy, +390862434742, +390862433523, email addresses: piero.ruscitti@univaq.it, pieroruscitti@live.com.

-Key words

Ferritin, inflammation, macrophages, adult onset Still's disease, macrophage activation syndrome.

Supplementary materials

Additional material 1. The effects of FeL on gene expressions of macrophages.

(A-L) qRT-PCR of FeH (A), FeL (B), IL-1β (C), IL-6 (D), IL-10 (E), IL-12 (F), TNF-α (G), TGF-β (H), VEGF (I), NLRP3 (L). The FeL stimulation of macrophages for 120 and 240min, does not change the gene expressions of FeH, FeL, IL-1β, IL-6, IL-10, TNF-α, IL-12, NLPR3, TGF-β, and VEGF. Any single dot, in the figure, represents the median of triplicate experiments.

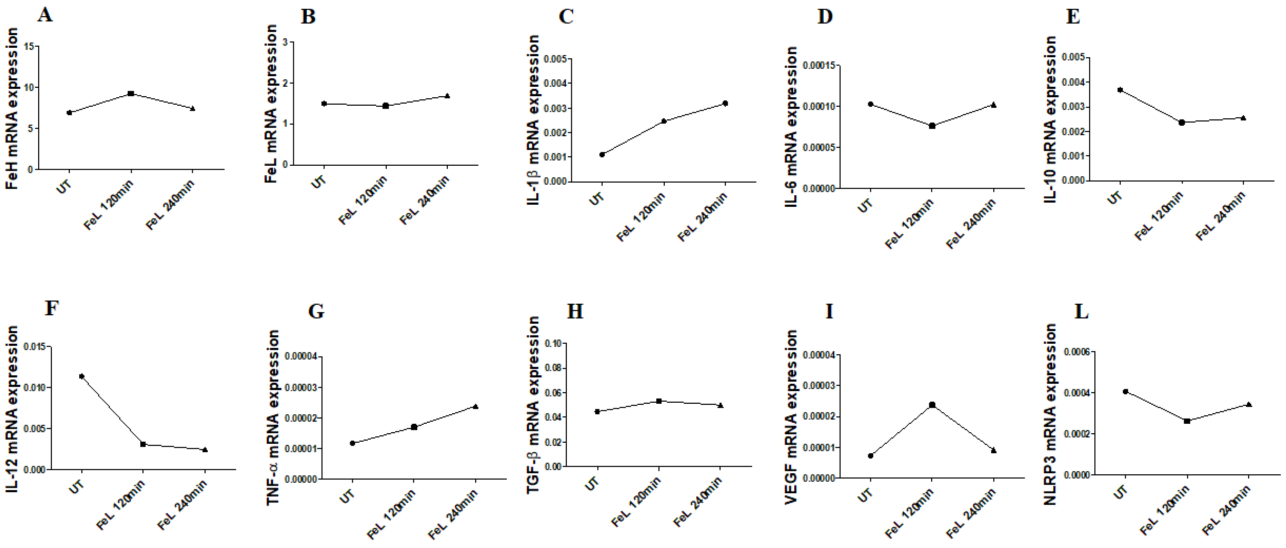

Additional material 2. Research Report Document: LC-MS/MS Identification of the Human H-Subunit of Ferritin in the Sera of Patients Affected by Inflammatory Conditions.

(A-J) Identification of the human H-subunit of ferritin in the sera of patients affected by inflammatory conditions.

Western blot analysis of human sera (Figure A) shows bands of interest in the pathologic sample (Red circles; Lane 5) which could be the H-subunit of ferritin when compared to the recombinant form of the protein of interest (Blue circle; Lane 2). LC-MS/MS analysis to determine identification was requested.

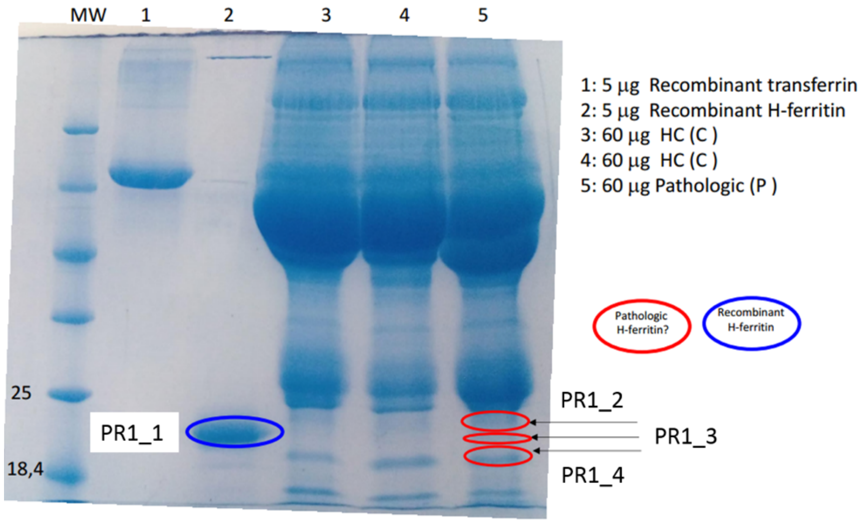

Figure A: SDS PAGE gel separation prior to digestion and protein identification with LC-MS/MS. Column 1 contains which human recombinant transferrin (1), human recombinant H-ferritin (2); sera of 2 human healthy controls (3,4), and human pathologic serum (5). The blue circle indicates the human recombinant commercial H-ferritin while the red circles indicate the 3 bands for analysis in the pathologic sample (molecular weights 18-25 kDa).

Methods

Enzymatic Digestion

In-gel reduction, alkylation and digestion with trypsin were performed on the four gel bands prior to subsequent analysis by mass spectrometry. Cysteine residues were reduced with dithiothreitol and derivatised by treatment with iodoacetamide to form stable carbamidomethyl derivatives. Trypsin digestion was carried out overnight at room temperature after initial incubation at 37oC for 2 hours.

LC-MS/MS

Peptides were extracted from the gel pieces by a series of acetonitrile and aqueous washes. The extract was pooled with the initial supernatant and lyophilised. Each sample was then resuspended in 10μL of 50mM ammonium bicarbonate and analysed by LC/MS/MS. Chromatographic separations were performed using an EASY NanoLC system (ThermoFisherScientific, UK). Peptides were resolved by reversed phase chromatography on a 75 μm C18 column using a three step linear gradient of acetonitrile in 0.1% formic acid. The gradient was delivered to elute the peptides at a flow rate of 300 nL/min over 120 min. The eluate was ionised by electrospray ionisation using an Orbitrap Velos Pro (ThermoFisherScientific, UK) operating under Xcalibur v2.2. The instrument was programmed to acquire in automated data-dependent switching mode, selecting precursor ions based on their intensity for sequencing by collision-induced fragmentation using a Top20 CID method. The MS/MS analyses were conducted using collision energy profiles that were chosen based on the mass-to-charge ratio (m/z) and the charge state of the peptide.

Table 1: LC/MS/MS reference table. Up = Uniprot database; AT = All Taxonomy; NP = In-house database

**Sample**	**LC/MS/MS Ref.**	**Date Analysed**	**Mascot Search Ref.**
PR1_1	SL20170508	08/05/2017	44813up AT; 45387np
PR1_2	SL20170508	08/05/2017	44812up AT; 45388np
PR1_3	SL20170508	08/05/2017	44814up AT; 45389np
PR1_4	SL20170508	08/05/2017	44815up AT; 45390np

Database Searching

Raw mass spectrometry data were processed into peak list files using Proteome Discoverer (ThermoScientific; v2.1) (Figure B).

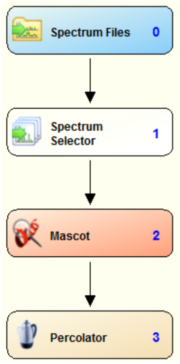

Figure B: Proteome discoverer nodal workflow for raw data processing

Processed raw data was searched using the Mascot search algorithm (v2.6; www.matrixscience.com) against the Uniprot database with All Taxonomy. The data was also searched against an in-house curated database containing only the H-ferritin protein of interest (Table 1).

Results

LC/MS/MS analysis has successfully identified both H-ferritin and L-ferritin along with many other proteins from the four sample bands. The samples were searched using All Taxonomy in Uniprot and also an in-house curated database containing the H-ferritin protein sequence only. Database generated files were uploaded into Scaffold 4 (v4.7.5) software (www.proteomesoftware.com) to create .sfd files (PR455 PieroRuscittiR1_1_1_4 20170519_EDIT; Figure C).

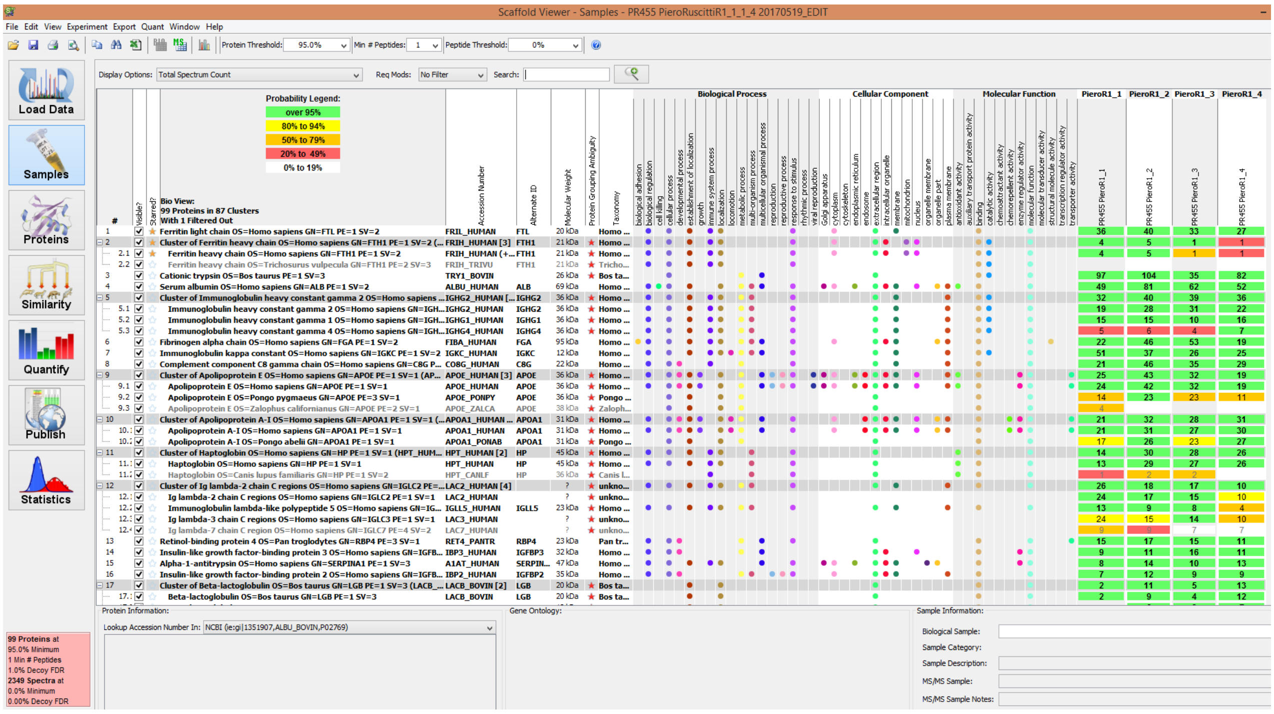

Figure C: Scaffold sample view representing protein identification from the four gel bands following database searching against All Taxonomy in the Uniprot database.

Scaffold allows statistical filtering of the data at the protein and peptide level. Mascot applies a 95% probability CI in the MOWSE scoring algorithm that is an identification threshold. This threshold is calculated as described on the Matrix Science website:

"Given an absolute probability that a match is random, and knowing the size of the sequence database being searched, it becomes possible to provide an objective measure of the significance of a result. A commonly accepted threshold is that an event is significant if it would be expected to occur at random with a frequency of less than 5%."

Any protein that is above this identity threshold is deemed significant.

Following searching against All Taxonomy at 95% CI probability, minimum of 1 peptide and 0% CI peptide probability, 50 proteins were detected in sample PR1_1. 65 proteins were detected in PR1_2, 57 proteins detected in PR1_3 and 80 proteins detected in PR1_4.

As the sample was a mixed population of proteins identified from each band, filtering of the data to highlight the protein of interest was performed. In the Scaffold software it is possible to filter the data using terms in the search box. In the first instance, typing 'ferritin' in to the search box highlights the proteins from the individual samples. Figure D represents the protein identification and total peptide counts for the protein in each band.

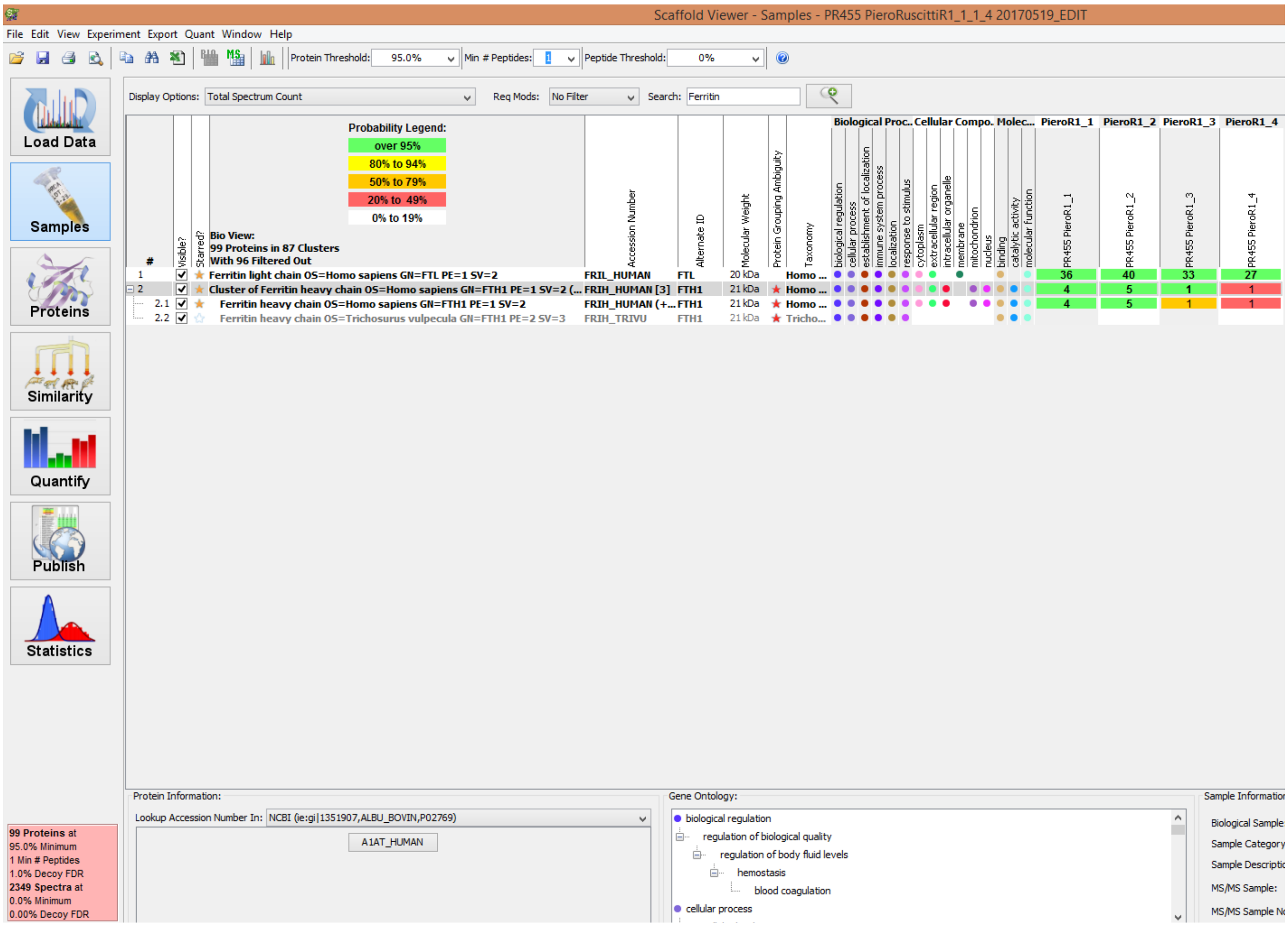

Figure D: Scaffold sample view following filtering for ferritin protein of interest

Although the sample was the heavy chain of ferritin (H-ferritin), especially the recombinant sample PR1_1, the best matched search from the database was for the light chain form, L-ferritin. Figures E-H represent the sequence coverage of L-ferritin for each individual band.

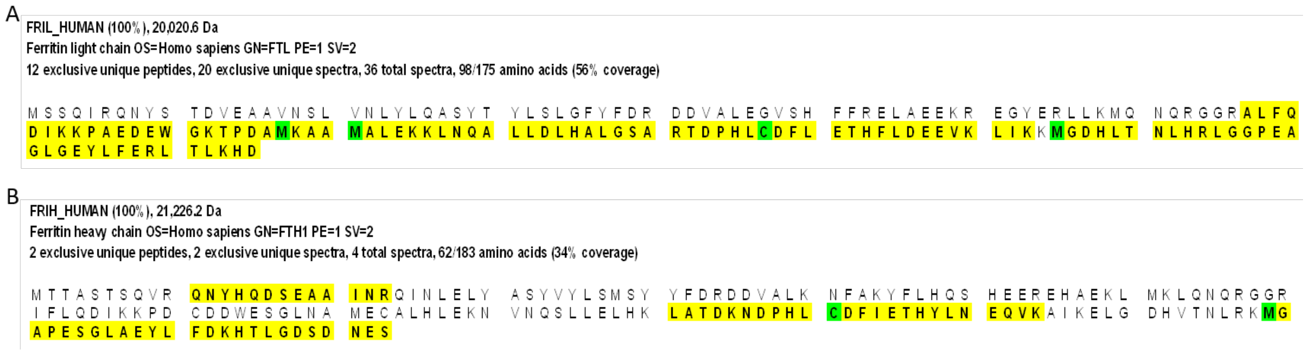

Figure E: Sequence coverage of the light chain form of ferritin (panel A) and the heavy chain form (panel B) from the recombinant band sample

PR1_1.

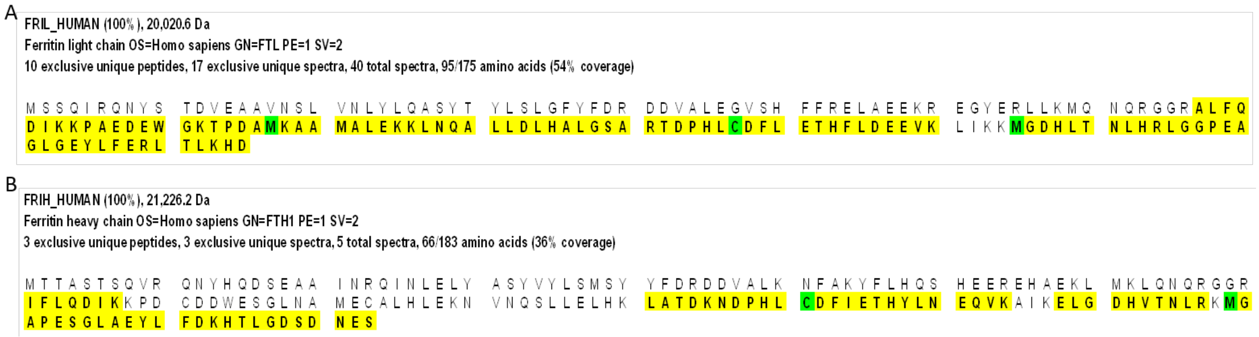

Figure F: Sequence coverage of the light chain form of ferritin (panel A) and the heavy chain form (panel B) from the recombinant band sample PR1_2.

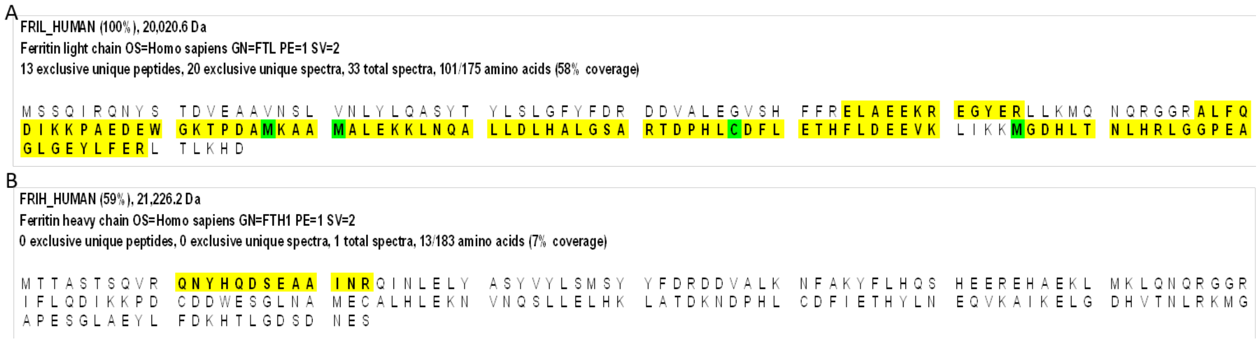

Figure G: Sequence coverage of the light chain form of ferritin (panel A) and the heavy chain form (panel B) from the recombinant band sample PR1_3.

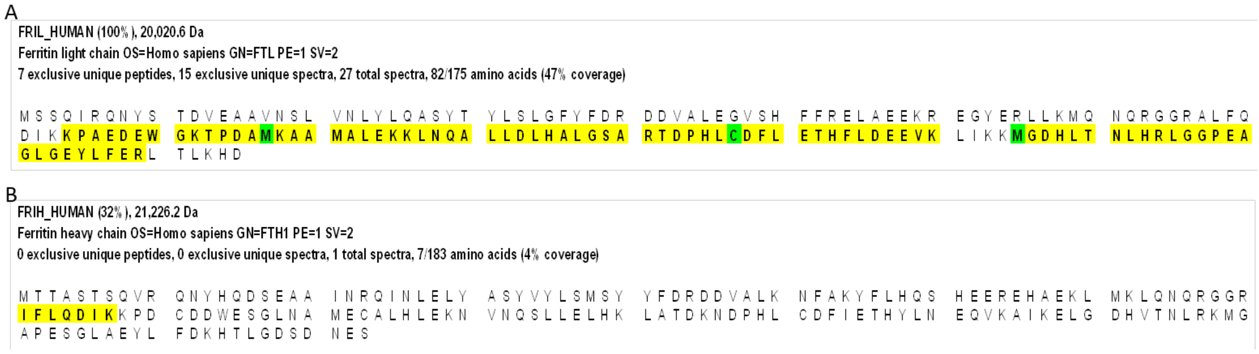

Figure H: Sequence coverage of the light chain form of ferritin (panel A) and the heavy chain form (panel B) from the recombinant band sample PR1_4.

Following database analysis against All Taxonomy in Uniprot, the sequence coverage of the light chain form is consistent across the four samples and

ranges from 47-58% coverage. The amount of total peptides detected in each sample varies from 27 to 36. The total number of peptides detected is an indicator of how abundant the protein is within the sample however it should only be used as a relative measure when comparing it to the sample protein across the rest of the samples. With this in mind, the amount of peptides matching to the sequence of the heavy chain form is very low which was not expected.

The database algorithm tries to match unknown processed peptide ions to a protein sequence by probability. A weighting of the identifications can occur if the algorithm deems that a certain protein is matched more significantly than another isoform of the same protein. To see if this had an effect on this dataset, an in-house database was curated containing only the FASTA file of the H-ferritin protein of interest and searched again (Figure I).

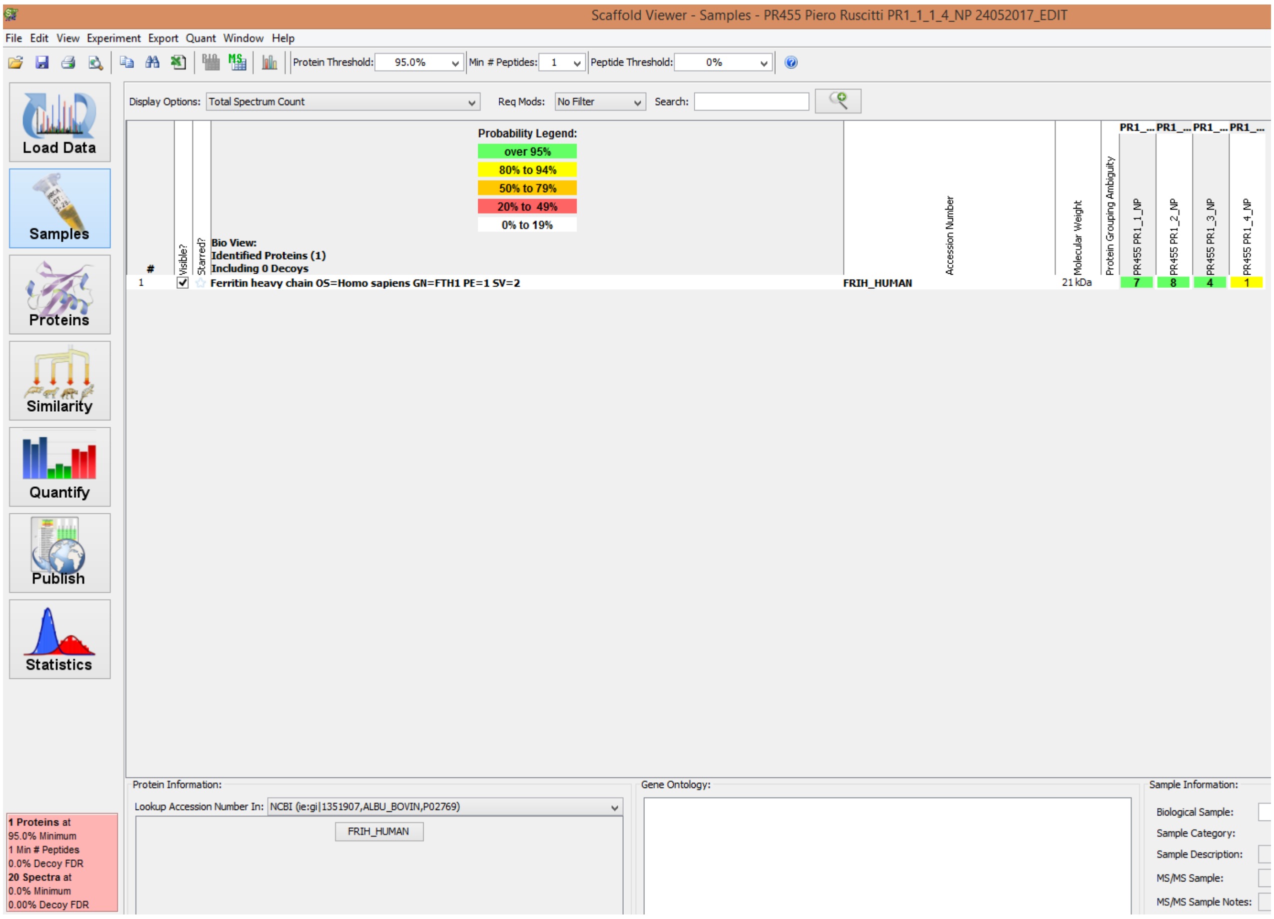

Figure I: Scaffold sample view following searching against a bespoke curated database containing only the H-ferritin sequence.

The new search was uploaded in to a separate Scaffold file for analysis (PR455 PR1_1_1_4_NP 24052017_EDIT). This new search increased the amount of peptides matched to the H-ferritin form when compared to the Uniprot search but it is still not as convincing as the peptide matching to the L-ferritin form (Fig J). This suggests that the protein being detected in the pathological sample is more likely to be the light-chain form after probability matching at 95% CI however, this should not be the true of the recombinant protein sample PR1_1 as it was specifically the H-ferritin protein form.

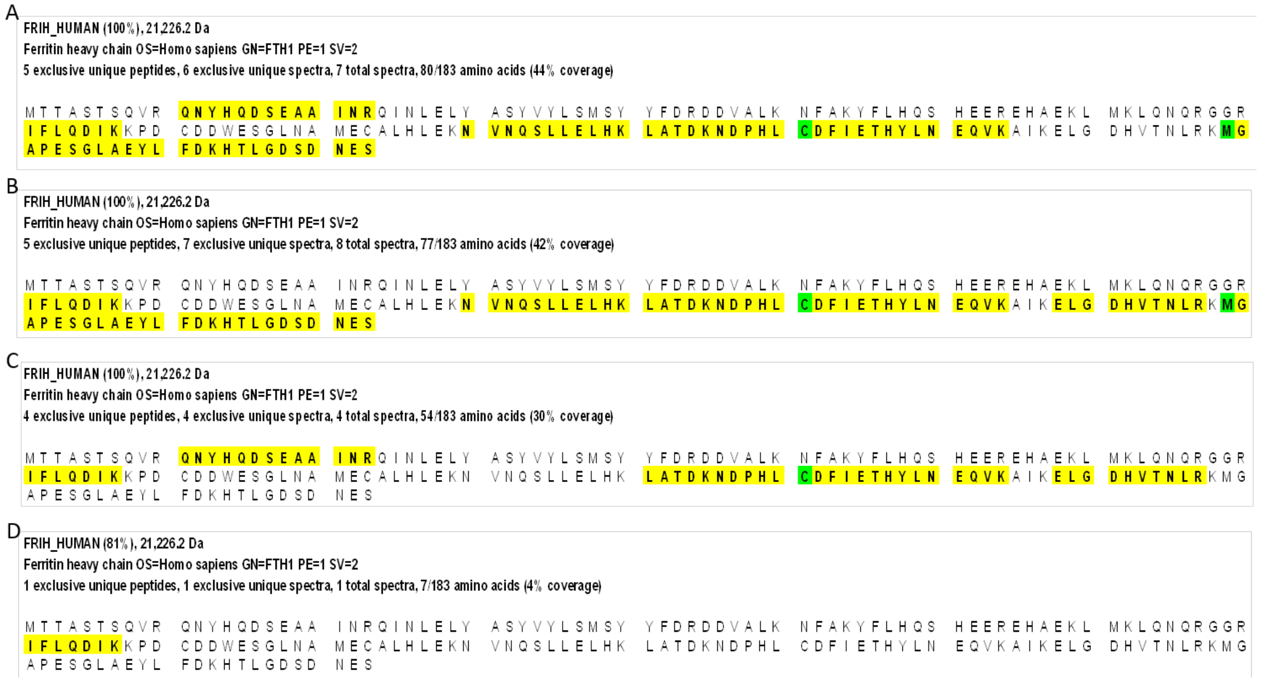

Figure J: Sequence coverage following searching against the H-ferritin in a curated database containing only the sequence of interest

Report Conclusions

LC/MS/MS has successfully sequenced and identified ferritin in both H- and L-forms from the four 1D SDS PAGE gel bands, the biological relevance of which can now be further explored. Database searching suggests that the dominant form after stringent probability matching is the light chain L-ferritin form and not the expected heavy chain H-ferritin form.

Additional material 3. Characteristics of monocytes, before and after M-CSF stimulation.

(A)Immunofluorescence staining of CD14 and CD68 in monocytes before M-CSF stimulation. The picture shows that the percentage of CD14+ cells is higher when compared to the percentage of CD68+ cells. (B) Immunofluorescence staining of CD14 and CD68 in macrophages after 7 days of M-CSF stimulation. The picture shows that the percentage of CD14+ cells is lower when compared with the percentage of CD68+ cells. Pictures are representative of all experiments. Original magnification 20X.

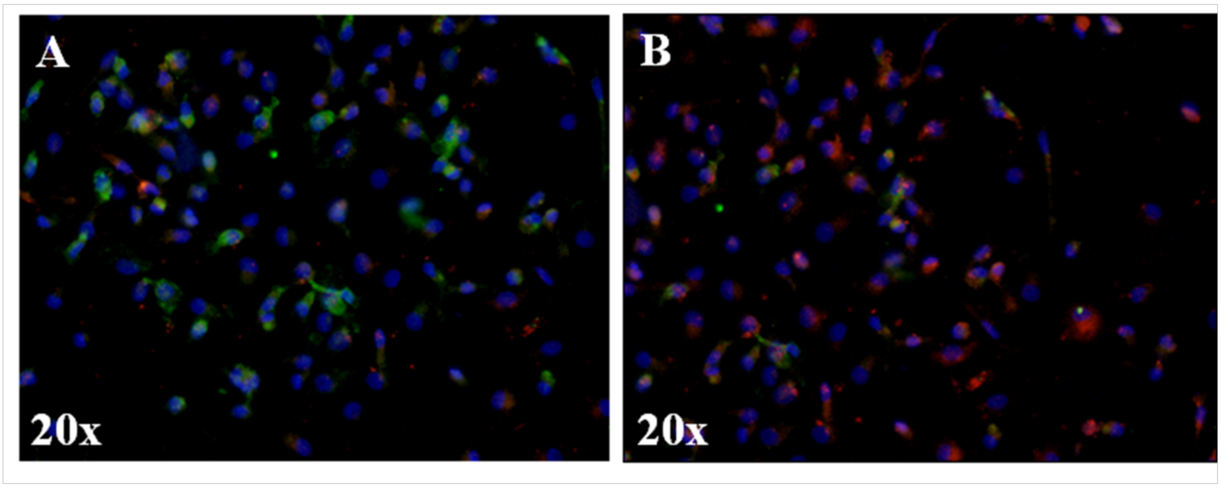

Additional material 4. Full-length blots of triplicate experiments assessing the effects of ferritin on protein expressions of macrophages.

(A-D) Western blot derived from the first experiment. Full-length blots of the same gel, showing IL-1β (A) 35 kDa, β-actin (ACT) (B) 43 kDa, NLRP3 (C) 110kDa and IL-12 (D) 70kDa.

(E-I) Western blot derived from the second experiment. (E-G) Full-length blots of the same gel, showing IL-1β (E) 35 kDa, ACT (F) 43 kDa and IL-12 (G) 70kDa. (H-I) Full-length blots of the same gel, showing NLRP3 (H) 110 kDa and ACT (I) 43 kDa.

(J-N) Western blot derived from the third experiment. (J-L) Full-length blots of the same gel, showing IL-1β (J) 35 kDa, ACT (K) 43 kDa and IL-12 (L) 70kDa. (M-N) Full-length blots of the same gel, showing NLRP3 (M) 110kDa and ACT (N) 43 kDa.

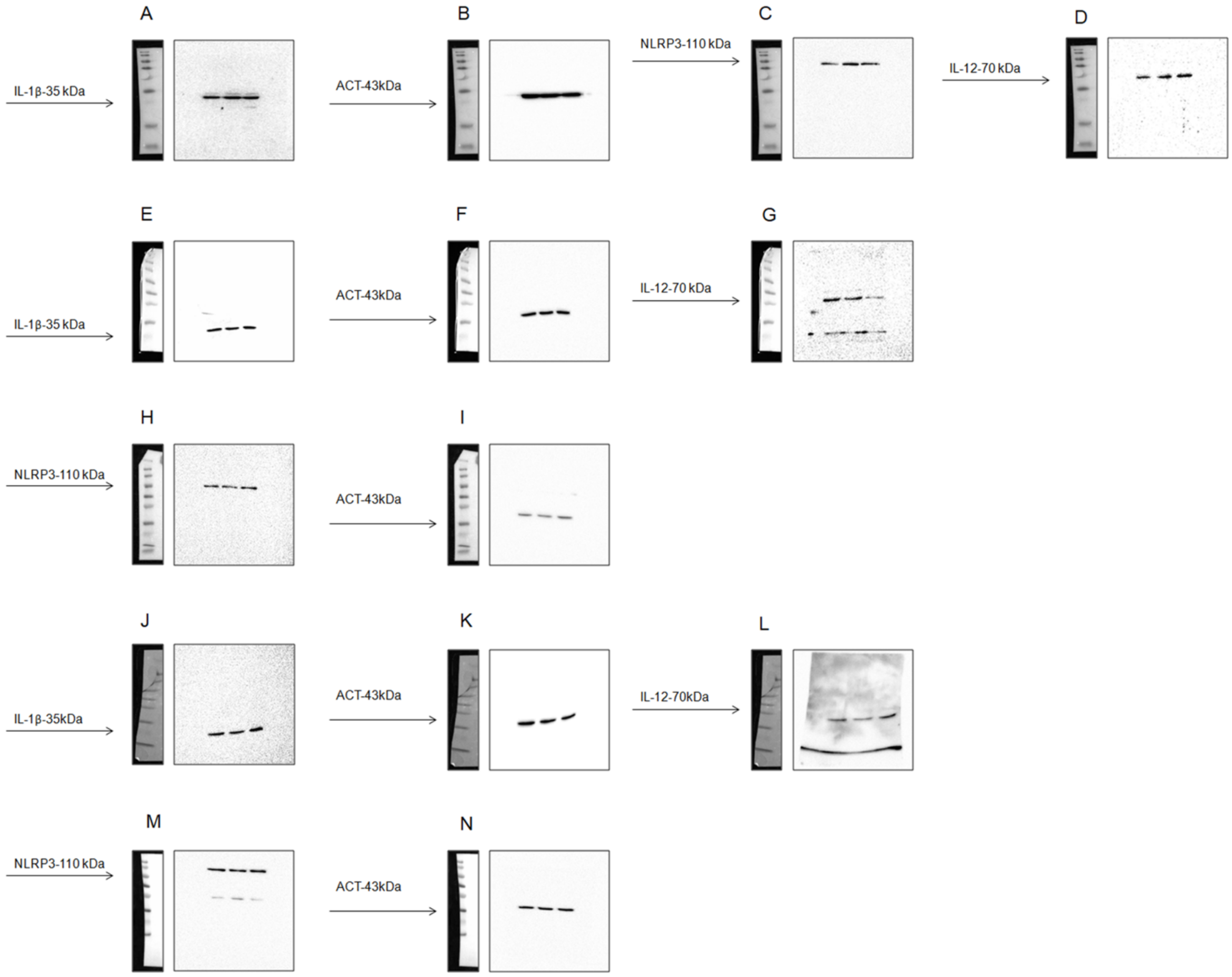

Additional material 5. Full-length blots of triplicate experiments assessing the effects of FeH on protein expressions of macrophages.

(A-D) Western blot derived from the first experiment. Full-length blots of the same gel, showing IL-1β (A) 35 kDa, ACT (B) 43 kDa, NLRP3 (C) 110kDa and IL-12 (D) 70kDa. In the full-length blots B, the signal of IL-1β protein is also visible, because non-completely removed after stripping procedure.

(E-H) Western blot derived from the second experiment. Full-length blots of the same gel, showing IL-1β (E) 35 kDa, ACT (F) 43 kDa, NLRP3 (G) 110kDa and IL-12 (H) 70kDa. In the full-length blots F, the signal of IL-1β protein is also visible, because non-completely removed after stripping procedure.

(I-M) Western blot derived from the third experiment. (I-K) Full-length blots of the same gel, showing IL-1β (I) 35 kDa, β-actin (ACT) (J) 43 kDa and IL-12 (K) 70kDa. In the full-length blots J, the signal of IL-1β protein is also visible, because non-completely removed after stripping procedure. (L-M) Full-length blots of the same gel, showing NLRP3 (L) 110kDa and β-actin (ACT) (M) 43 kDa.

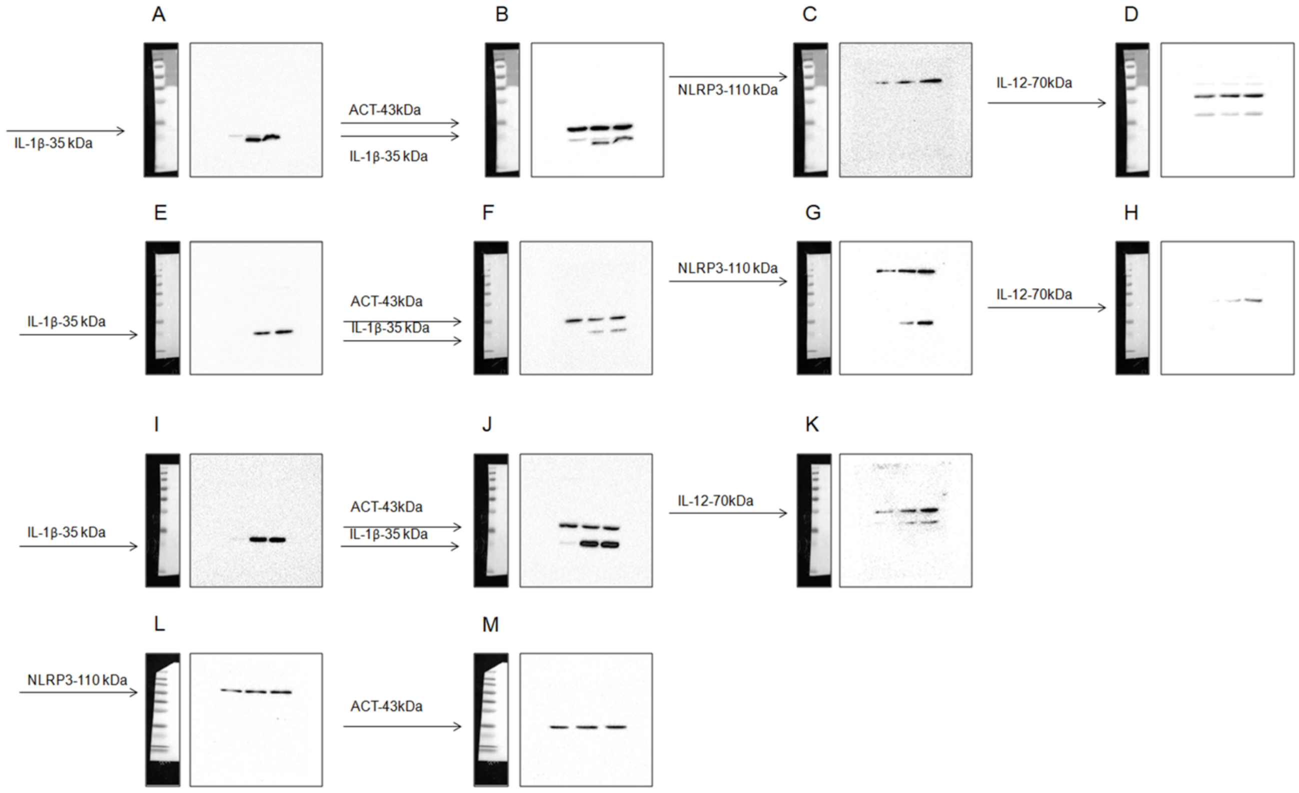

<u>Additional material 6. The effects of Ferritin on gene expressions of macrophages.</u>

(A-L) qRT-PCR of FeH (A), FeL (B), IL-1β (C), IL-6 (D), IL-10 (E), IL-12 (F), TNF-α (G), TGF-β (H), VEGF (I), NLRP3 (L). Any single dot, in the figure, represents the mean±SEM of triplicate experiments.

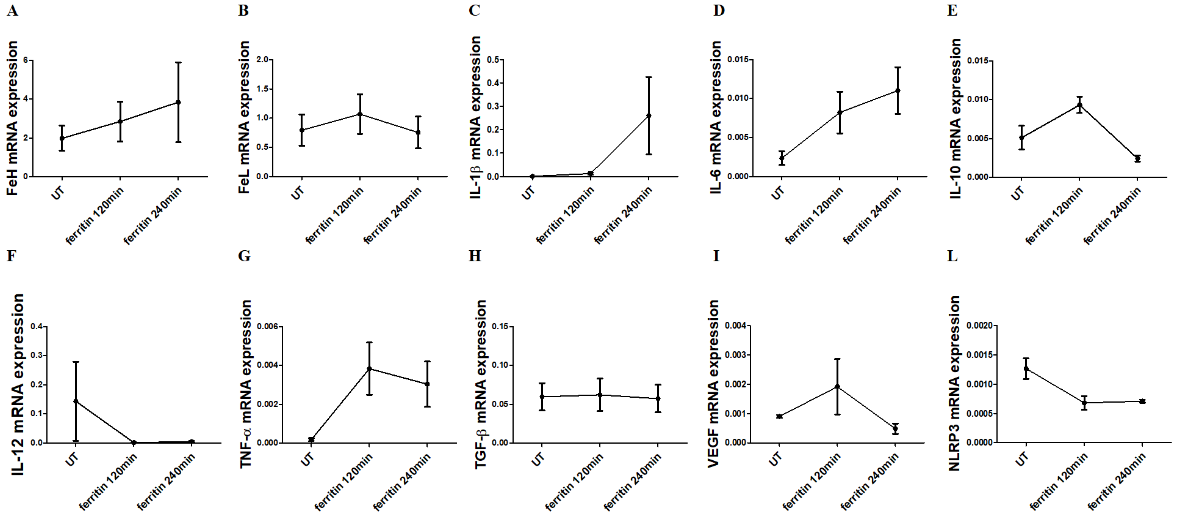

Additional material 7. The effects of FeH on gene expressions of macrophages.

(A-L) qRT-PCR of FeH (A), FeL (B), IL-1β (C), IL-6 (D), IL-10 (E), IL-12 (F), TNF-α (G), TGF-β (H), VEGF (I), NLRP3 (L). Any single dot, in the figure, represents the mean±SEM of triplicate experiments.

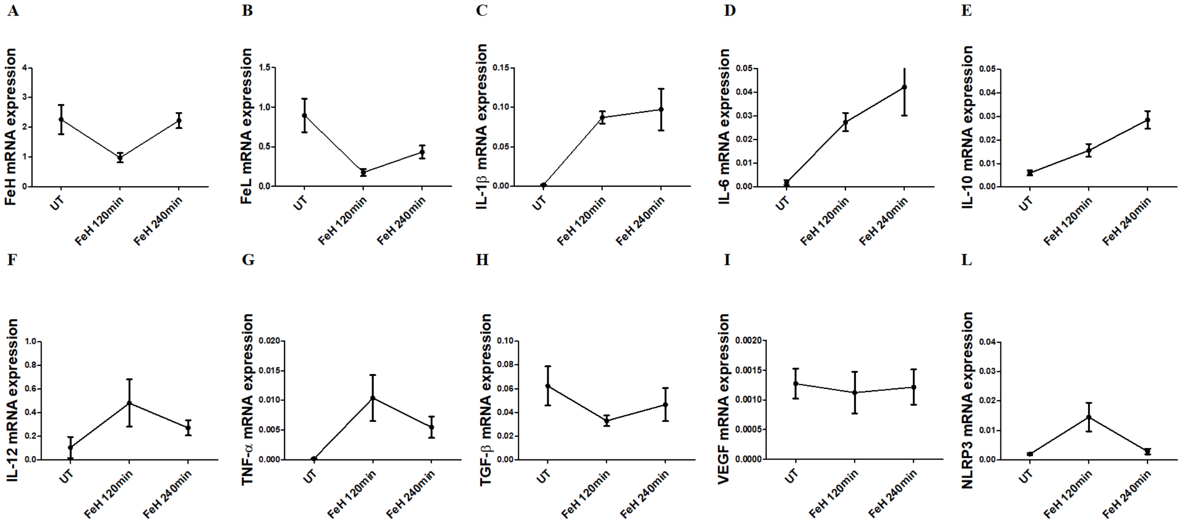

__Additional material 8. The effects of FeL on gene expressions of macrophages.__

(A-L) qRT-PCR of FeH (A), FeL (B), IL-1β (C), IL-6 (D), IL-10 (E), IL-12 (F), TNF-α (G), TGF-β (H), VEGF (I), NLRP3 (L). Any single dot, in the figure, represents the mean±SEM of triplicate experiments.

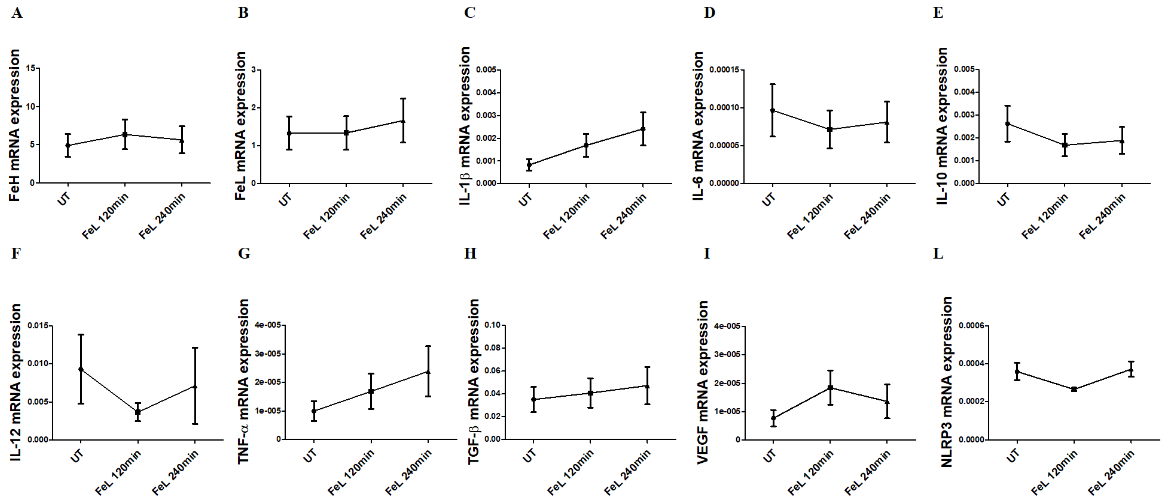

Supplement: Supplementary file 1 — Supplementary Information [file 41598_2020_69031_MOESM1_ESM.pdf]
